# Vicarious body maps bridge vision and touch in the human brain

**DOI:** 10.1038/s41586-025-09796-0

**Published:** 2025-11-26

**Authors:** Nicholas Hedger, Thomas Naselaris, Kendrick Kay, Tomas Knapen

**Affiliations:** 1 Centre for Integrative Neuroscience and Neurodynamics, University of Reading, Reading, UK; 2 Center for Magnetic Resonance Research, Department of Radiology, University of Minnesota, Minneapolis, MN USA; 3 Department of Neuroscience, University of Minnesota, Minneapolis, MN USA; 4 Spinoza Centre for Neuroimaging, Amsterdam, The Netherlands; 5 Department of Computational Cognitive Neuroscience and Neuroimaging, Netherlands Institute for Neuroscience, Amsterdam, The Netherlands; 6 Department of Experimental and Applied Psychology, Vrije Universiteit Amsterdam, Amsterdam, The Netherlands

**Keywords:** Sensory processing, Extrastriate cortex

## Abstract

Our sensory systems work together to generate a cohesive experience of the world around us. Watching others being touched activates brain areas representing our own sense of touch: the visual system recruits touch-related computations to simulate bodily consequences of visual inputs^1^. One long-standing question is how the brain implements this interface between visual and somatosensory representations^2^. Here, to address this question, we developed a model to simultaneously map somatosensory body part tuning and visual field tuning throughout the brain. Applying our model to ongoing co-activations during rest resulted in detailed maps of body-part tuning in the brain’s endogenous somatotopic network. During video watching, somatotopic tuning explains responses throughout the entire dorsolateral visual system, revealing an array of somatotopic body maps that tile the cortical surface. The body-position tuning of these maps aligns with visual tuning, predicting both preferences for visual field locations and visual-category preferences for body parts. These results reveal a mode of brain organization in which aligned visual–somatosensory topographic maps connect visual and bodily reference frames. This cross-modal interface is ideally situated to translate raw sensory impressions into more abstract formats that are useful for action, social cognition and semantic processing^3^.

## Abstract (web summary)

A mode of brain organization that connects visual and bodily reference frames may translate raw sensory impressions into more abstract formats that are useful for action, social cognition and semantic processing.

## Main

In humans, input from one sensory modality can influence processing in another, enriching perception and understanding. One notable case is how visual inputs recruit somatosensation, our sense of touch: when seeing others in pain, we may wince and even remark that we ‘felt their pain’. Indeed, when observing others, our brain often responds ‘as if’ their tactile experience were our own. Human neuroimaging work has demonstrated extensive, body-part-specific activations in motor and somatosensory cortex when participants view others acting on objects^4^ or being touched^2^. This cross-modal relationship between vision and touch is bidirectional: regions of high-level visual cortex similarly exhibit body-part-specific activations when participants perform unseen actions^5,6^. These ‘foreign source’ activations of our sensorimotor and visual systems have been linked to vicarious and empathic experiences^1,7^. Despite the pervasive nature of these intermodal activations, we lack understanding of how the brain connects the computational machinery of vision and somatosensation.

W. James proposed that the body is a constant participant in cognition, preventing mental content from ever being “purely disembodied”^8^. Indeed, in addition to being engaged by input from vision, our somatosensory system is likely to be engaged endogenously—spontaneous thought is often self-referential, involving body-referenced processes of emotional introspection, interoception and proprioception^9,10^. Accordingly, this predicts the engagement of unimodal somatosensory brain regions even in the absence of any exogenous sensory stimulation. By contrast, when somatosensation is recruited by visual inputs, our brains must recode visual signals into somatosensory representations. This predicts that visual inputs should engage multimodal neural sites with selective tuning tethered to both visual and somatosensory reference frames. This putative joint visual–touch tuning aligns with models of ‘embodied’ visual perception, which view visual perception as inherently multisensory and shaped by potential bodily interactions^11^. A further testable prediction flows from this idea: neural responses to visual input affording bodily interactions should be better explained by models incorporating selective tuning in both visual and somatosensory modalities, rather than visual tuning alone. While these normative predictions are theoretically well founded, their validation is hindered by the lack of an explicit computational model that captures multimodal tuning across endogenous and non-afferent conditions. Here we introduce such a model and apply it to directly test these predictions.

After testing such predictions, a key question remains: what rules govern the relationship between somatosensory and visual tuning? One possibility is alignment to environmental statistics, with the visual system’s retinal reference frame imposed on somatosensation to match ecological regularities in body-part positions^3^. For example, somatosensory tuning to foot sensations may predict visual tuning to lower visual field locations in which feet typically appear. Another hypothesis posits a more category-selective level of alignment: somatosensory selectivity for foot sensations may predict visual selectivity for presentations of feet, independent of their spatial location.

Here we show that modelling the topographic structure of naturalistic brain activations reveals the computational motifs connecting visual and touch representations. We show that structured connectivity to primary somatosensory cortex (S1) at rest predicted responses across large swathes of cortex. During video watching, this somatosensory network extended deeply into visual cortex. There, we identified multiple visually evoked somatotopic maps of which the tuning aligned with both visual field positions and visual body-part preferences. These cross-modal topographies may constitute a common language for sharing structured information between sensory modalities, supporting cohesive perception and cognition.

## Endogenous somatotopic maps

**Fig. 1:**
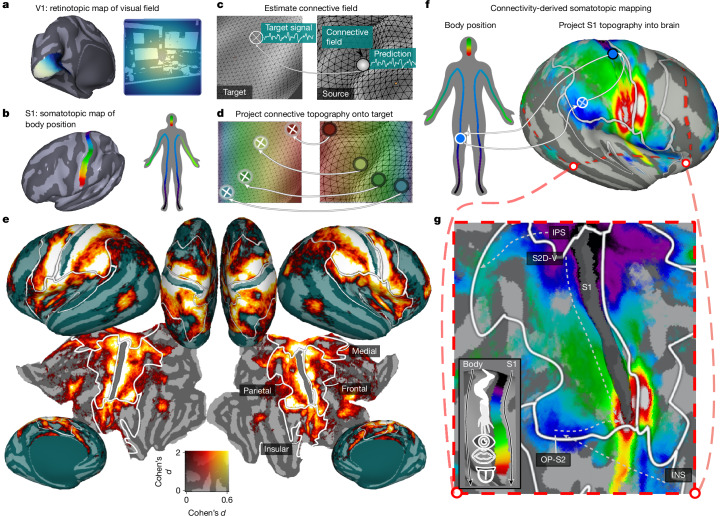
Connective-field modelling reveals endogenous somatotopic network. **a**,**b**, Schematic of the retinotopic organization of human V1 (**a**) and the somatotopic organization of S1 (**b**). Neighbouring positions in V1 and S1 are associated with sensitivity to neighbouring positions in the visual field and body respectively (colours). **c**, Connective-field modelling. A time-varying response within a target region (for example, higher-level somatosensory cortex) is modelled as some combination of signals (that is, a connective field) deriving from a source region (such as S1). **d**, With connective fields estimated for every part of the target region, the topographic map of the source can be projected onto the target according to the connectivity profile (colours). **e**, The strength of somatotopic connectivity during resting state on the cortical surface, expressed as Cohen’s *d*_*z*_. The white borders indicate the boundaries of gross-anatomical somatosensory regions denoted by the labels. **f**, Somatotopic mapping. Spatial connectivity with S1 can be leveraged to project the S1 somatotopic map into the rest of the brain, revealing connectivity-derived somatotopic structure from resting-state data. **g**, The same data, but for the region in **f** demarcated by the red outline. Data are thresholded to show vertices with significant somatotopic connectivity (*n* = 174, *α* = 0.05). These surface visualizations are presented thresholded without correction for multiple comparisons, but all regions analysed further survive cluster-based correction (see the ‘Thresholding of model performance’ section in the Methods). The arrows indicate the location of homuncular gradients described in the main text. The S1 source region (the data underlying the design matrix for our modelling) is removed so that only target data are shown.

**Extended Data Fig. 1:**
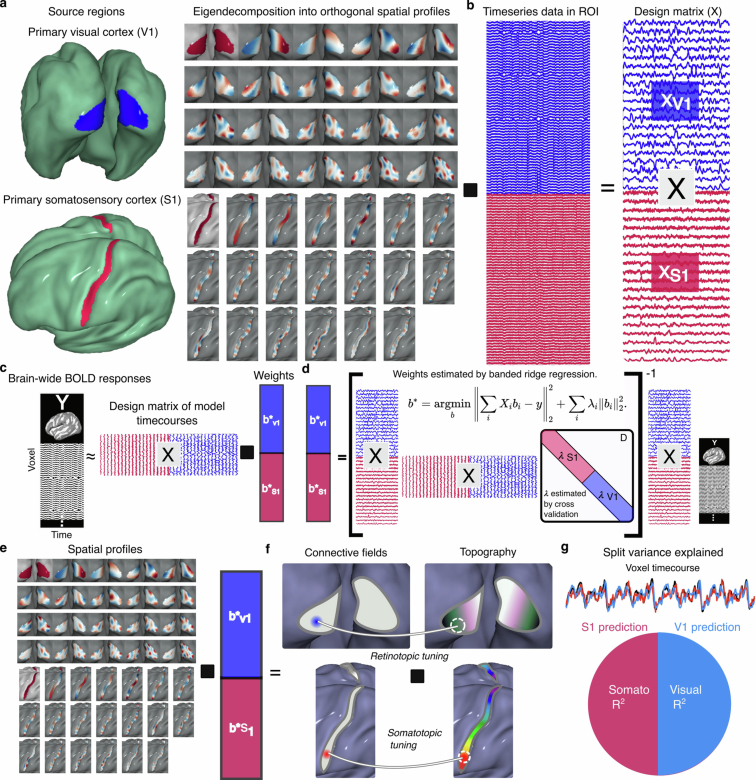
Multisensory connective field modelling procedures. **a**. Initial source regions are defined from the vertices corresponding to V1 and S1, from which 200 LBOEs are defined. **b**. The dot product of these spatial profiles and the BOLD timecourses for each vertex within each subsurface produces a banded design matrix of model timecourses. **c**. For each voxel in the brain, its BOLD timecourse is estimated as a linear combination of the model timecourses in the design matrix. **d**. Parameters of the GLM are estimated by banded ridge-regression, employing a leave-one-run-out cross-validation scheme. **e**. With ridge beta weights (b*) estimated, the connective field for V1 and S1 is estimated as the dot product of the b* and the corresponding LBOEs. **f**. The retinotopic and somatotopic tuning of each voxel is then estimated as the weighted average of its estimated connective field profiles and the topographic map of each source region. **g**. The dual, banded nature of the design matrix allows the variance explained to be partitioned into the split proportion explained by V1 and S1 connective fields. These split *R*^2^ values sum to the total variance explained. See Methods for more details.

**Extended Data Fig. 2:**
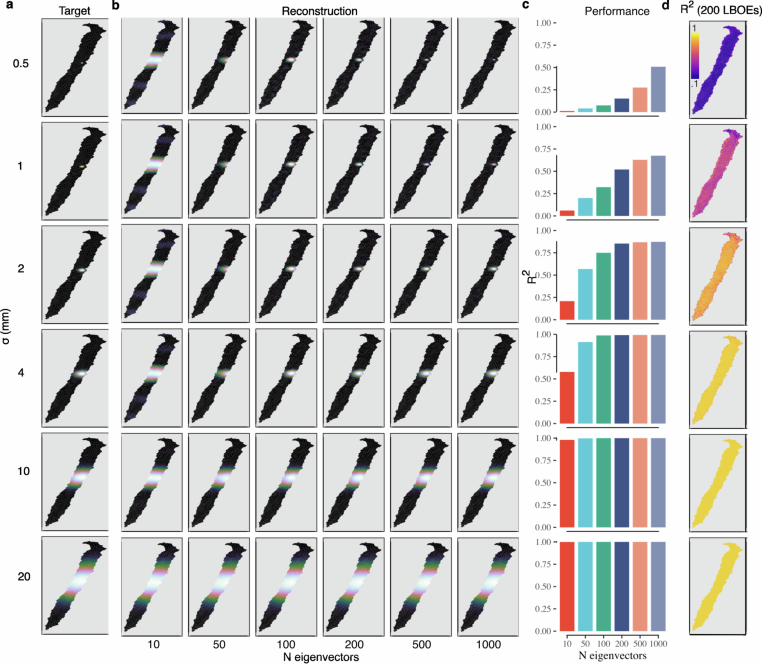
Validation of connective field estimation: S1. **a**. Depicts a target connective field on the surface of S1, defined by a Gaussian function with a standard deviation (σ) defined in mm. **b**. The predicted connective field as a function of the number of S1 LBOEs in the design matrix. **c**. The agreement (defined by *R*^2^) between the predicted and target connective fields as a function of *σ* and number of LBOEs. **d**. For each vertex in S1, depicts the *R*^2^ for a model with 200 LBOEs.

**Extended Data Fig. 3:**
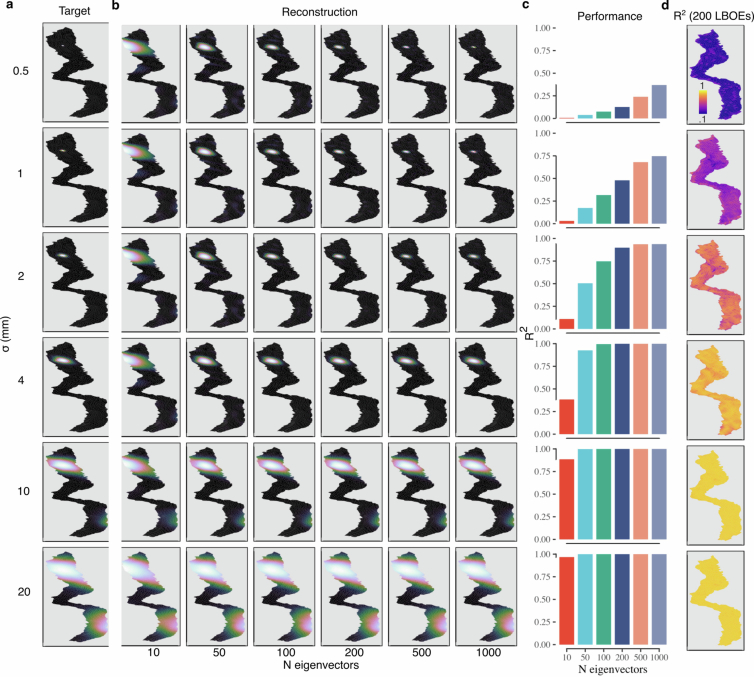
Validation of connective field estimation: V1. **a**. Depicts a target connective field on the surface of V1, defined by a Gaussian function with a given standard deviation (σ). **b**. The predicted connective field as a function of the number of V1 LBOEs in the design matrix. **c**. The agreement (defined by *R*^2^) between the predicted and target connective fields as a function of σ and number of LBOEs. **d**. For each vertex in V1, depicts the R2 for a model with 200 LBOEs.

We developed a computational model that capitalizes on a shared principle of our sensory systems: topographic organization. Primary visual cortex (V1) contains a retinotopic map, with neighbouring regions tuned to neighbouring locations of the visual field^12^. Similarly, S1 contains a somatotopic map, with neighbouring locations tuned to neighbouring bodily locations^13^ (Fig. 1a,b). For every target voxel in the brain, we estimate the spatial patterns (connective fields^14^) on the source regions of V1 and S1 that best explain its blood-oxygenation-level dependent (BOLD) time-course (source region definitions are provided in the Methods). As patterns in these source regions relate directly to visual field and body positions, they allow connectivity-derived mapping of voxels’ visual and body-part tuning (Extended Data Figs. 1–3). This dual-source connective field effectively ‘projects’ the topography of the source regions into target regions, revealing neurally referred retinotopic and somatotopic maps (Fig. 1c,d).

The retinotopic organization of the visual system can be revealed from resting-state BOLD responses^15,16^, suggesting an endogenous sensory-topographic structure that is recruited during spontaneous thought. Similarly, resting state connectivity within S1 is organized by functional body-part boundaries^17,18^, implying that brain-wide topographic mapping of the somatosensory system may also be viable by leveraging resting state connectivity to S1. To test this, we fit our model to 1 h of 7 T resting-state data from each of 174 Human Connectome Project (HCP) participants.

Figure 1e shows the performance of S1 connective fields in predicting brain responses during rest, expressed as the degree of somatotopic connectivity (Methods). A large swath of cortex, encompassing classically defined somatosensory regions in parietal, frontal, medial and insular cortex exhibited somatotopic connectivity (all *P* < 10^−8^, minimum Cohen’s *d* = 0.41: Fig. 1f,g), with the strongest effects detected in parietal and frontal cortex (main effect of region of interest (ROI): *F*_2.5,431.69_ = 488.66, *P* < 10^−125^).

**Extended Data Fig. 4:**
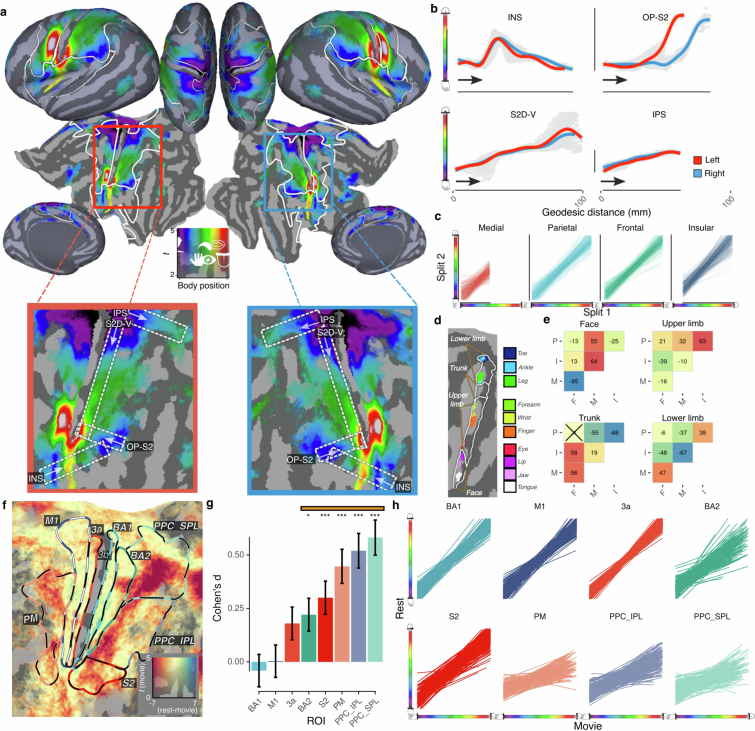
Structure and reliability of resting state somatotopy and comparison of movie-rest somatotopy. **a**. Shows preferred S1 position (colours) derived from connective field modelling of resting state data. **b**. For each hemisphere (colours) shows the relationship between S1 position and geodesic distance along each of the gradients (arrows) depicted in the lower panel of **a**. **c**. Lines represent least-squares fits for the relationship between somatotopic maps of independent subject splits. Different panels/colours show the data for different somatotopic rois, which correspond to the solid white lines in **a**. **d**. Depicts 4 topographic body part fields of 3b. Coloured patches on the 3b surface depict the location of the peak *t* statistics corresponding to the 10 body part movements (see legend) according to independent somatotopy data (see Methods). All lower limb and upper limb peaks fall within their corresponding field. The eye peak (red) sits on the border of the upper limb and face field and no peaks fall within the trunk field - since this was not represented in the somatotopy dataset. **e**. Depicts the between somatotopic ROI pairwise differences in connective field energy contained in each body part field. The values in each cell are signed log10 transformed *p* values. Larger values for a parameter in a reference ROI (y axis) compared to a comparison ROI (x axis) are assigned positive values (*p* values are -log10 transformed). Smaller values in the reference ROI are assigned negative values (*p* values are log10 transformed). *p* values that do not reach the holm-bonferroni corrected alpha level of .05 are marked by a cross in the corresponding cell (F = frontal, P = parietal, M= medial, I = insular). Tests are two sided. **f**. Shows the difference in somatotopic connectivity between rest and movie-watching. Coloured overlays are subdivisions of the extended somatosensory network (PM = premotor cortex, M1 = primary motor cortex, PPC = posterior parietal cortex, divided into superior (SPL) and inferior parietal lobule (IPL). BA1, BA2, 3a and 3b = Glasser atlas definitions of Brodmann corresponding areas). See Methods for ROI definitions. **g**. Depicts the magnitude (Cohen’s *dz*) of the increase in somatotopic connectivity from rest to movie for each of the ROIs defined in **e**. Asterisks indicate statistical significance (* <.05, ** <.01, *** <.001) BA1: *p* =.999, 3a: *p* = .056, M1: *p* = .999, BA2: *p* =.016, SII: *p* < 10^−3^, PPC-IPL: *p* < 10^−9^, PPC-SPL: *p* < 10^−9^. Error bars are +/− 1SE around Cohen’s *dz* (*N* = 174). Tests are two sided and holm-bonferroni corrected for multiple comparisons. The orange vertical line depicts regions previously reported to exhibit ‘vicarious’ responses. **h**. Depicts least-squares fits for the relationship between preferred S1 position derived from rest and movie. Lines are individual participant data.

With robust intrinsic somatotopic connectivity established, we next examined the underlying topographic structure of these activations. By translating the connective-field profiles into S1 somatotopic map positions and projecting these into the brain (Fig. 1f), we observed several orderly somatotopic gradients that mirror classical hallmarks of human somatosensory organization^19,20^ (Fig. 1g): one dorsal-to-ventral toe–tongue gradient along the central sulcus (S2D-V), one posterior–anterior from the parietal operculum/S2 (OP-S2), a double gradient in insular cortex (INS) and an additional anterior–posterior gradient along the intraparietal sulcus (IPS). The somatotopic structure was replicated in independent participant splits (parietal, mean Fisher’s *z* (*M*_*z*_) = 0.99, 95% confidence intervals (CI) = 0.97–1.02; frontal, *M*_*z*_ = 1.08, 95% CI = 1.06–1.11; medial, *M*_*z*_ = 0.59, 95% CI = 0.56–0.62; insular, *M*_*z*_ = 0.81, 95% CI = 0.79–0.84; Extended Data Fig. 4).

A further hallmark of somatotopic maps is their biased representations of body parts, putatively reflecting functional specialization^13^. In S1, functional boundaries between body-part representations are thought to be reflected in distinct subfields, identifiable from myelin and resting-state connectivity gradients^17^. To examine how connectivity captures these representational biases, we analysed the proportion of connectivity to each of these subfields (Methods and Extended Data Fig. 4). This revealed distinct biases mirroring previous observations: medial regions had the largest proportion of lower-limb/trunk-tuned locations (maximum *P* for pairwise comparisons, 10^−37^), consistent with lesion evidence in leg-related pathology^21^. By contrast, the largest proportion of upper-limb-tuned locations was observed in parietal cortex (maximum *P* = 10^−21^), aligning with its role in reaching and grasping actions^22^.

The anatomical loci, extent and tuning properties of our somatotopic maps mirror those typically obtained from studies that use tactile or motor interactions^13,19^. However, crucially, our model demonstrates the intrinsic nature of this organization by establishing these principles under conditions lacking such external sensorimotor stimulation.

## Video viewing recruits the touch network

Our resting-state analyses revealed widespread intrinsic somatotopic organization mirroring patterns evoked by somatosensory stimulation. However, BOLD fluctuations during rest occur during unconstrained mentation that varies across individuals and time^23^, making it challenging to anchor them to specific mental content. To examine how our somatotopic network is influenced by structured naturalistic visual inputs, we next performed our connective-field modelling on 1 h of 7 T data obtained from the same participants during video watching.

**Fig. 2:**
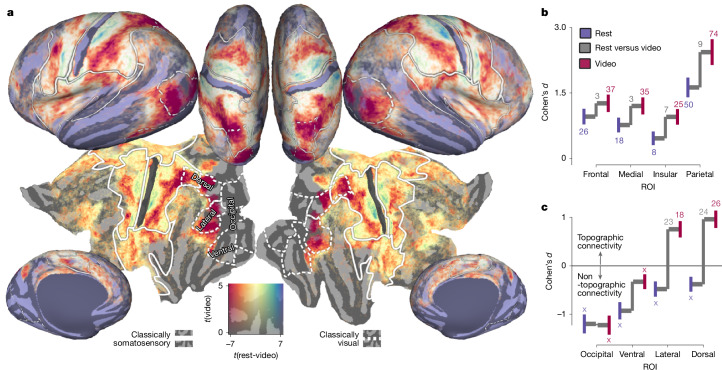
Somatotopic connectivity in visual cortex during video watching. **a**, Contrast of somatotopic connectivity between rest and video watching (red, greater during video watching; blue, greater during rest). The solid overlays indicate the somatotopic regions outlined previously, and the dotted lines indicate gross divisions of conventionally defined visual cortex (Methods). **b**, Throughout the somatosensory network, video watching increases the strength of somatotopic connectivity. The coloured vertical lines indicate the 95% confidence range of the estimate. The connecting grey lines illustrate the difference between conditions. Coloured and grey numbers indicate the −log_10_-transformed *P* value for the one-sample difference from 0 and the paired difference between task conditions respectively. *n* = 174. Tests were two-tailed and *P* values were Holm–Bonferroni corrected. An ‘X’ indicates conditions of non-topographic connectivity, where the null (nonspatial) model has superior generalization performance compared with the connective-field model. **c**, Focusing on the increased somatotopic connectivity in the visual system, we find that this connectivity is increased selectively in the lateral and dorsal portions of the visual system.

**Extended Data Fig. 5:**
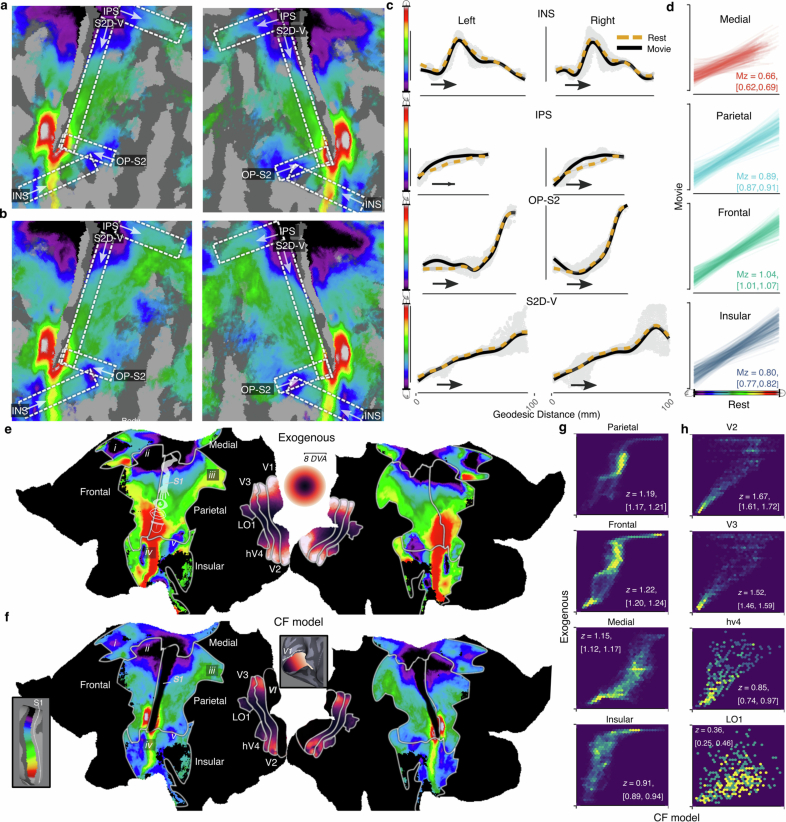
Comparison between rest, movie-watching and exogenously-derived maps. **a**. Shows preferred S1 position (colours) derived from connective field modelling of resting state data (same data as Extended Data Fig. 4a). **b**. For comparison, the same outcomes are plotted from the movie-watching data. **c**. For each hemisphere and task (rest, movie) depicts the relationship between S1 position and geodesic distance along each of the gradients depicted **a-b**. Points represent data from individual vertices. **d**. Lines represent least-squares fits for the relationship between somatotopic maps of rest and movie watching data from each subject. The text in each panel indicates the mean Fisher-z transformed correlation and 95% CI. **e**. Depicts exogenously-derived somatotopic and retinotopic maps for four somatotopic and retinotopic regions of the visual system. Eccentricity data (colorwheel) is derived from the ‘retinotopic prior’ derived from the HCP dataset and somatotopy data is derived from an independent whole brain somatotopy dataset described in Methods. Body part schematics are superimposed on S1 to indicate the location of peak preferences for each body part. **f**. The connective field model derived retinotopic and somatotopic maps are shown for the movie-watching data. Numerals are shown in **e-f** to highlight corresponding points. Detailed somatotopic structure of the exogenously-derived data is replicated by the connective field model. This is even true of insular and medial cortex where connective field model performance is comparatively low. **g-h**. Hexbin plots depicting the agreement between the connective field model-derived and exogenously-derived somatotopic and retinotopic maps. The text in each panel indicates the mean Fisher-z transformed correlation and 95% CI. All data in this figure are thresholded to only include vertices for which significant topographic connectivity score for the corresponding modality was detected (see Methods).

Figure 2a depicts the difference in somatotopic connectivity between rest and video watching. No differences were detected in primary areas closely tied to first person sensorimotor experience (Brodmann area 1 (video − rest): *t*_173_ = −0.54, *P* = 0.999, *d* = −0.01, 95% CI = −0.15–0.15; area 3a: *t*_173_ = 2.37, *P* = 0.056, *d* = 0.16, 95% CI = −0.03–0.30; primary motor cortex (M1): *t*_173_ = 0.04, *P* = 0.999, *d* = 0.01, 95% CI = −0.15–0.15). Moreover, detailed somatotopic organization was similar during video watching (Extended Data Figs. 4h and 5a–c) with strong correlations between preferred S1 position in video and rest (explicit statistics are provided in Extended Data Fig. 5d). However, relative to rest, a much larger swathe of half of cerebral cortex (50%, 95% CI = 46–55%) exhibits somatotopic connectivity during video watching (Fig. 2b,c). As neither rest or video watching involve exogenous sensorimotor stimulation, this implies cross-modal modulation of topographic connectivity whereby visual stimulation alone leads to more spatially organized activity in the somatosensory system. Increased somatotopic connectivity was detected in Brodmann area 2 (*t*_173_ = 2.91, *P* = 0.016, *d* = 0.18, 95% CI = 0.03–0.33), SII (*t*_173_ = 3.96, *P* < 10^−3^, *d* = 0.27, 95% CI = 0.12–0.42) and posterior parietal cortex, including the inferior parietal lobule (PPC–IPL) (*t*_173_ = 6.86, *P* < 10^−9^, *d* = 0.49, 95% CI = 0.34–0.65) and superior parietal lobule (PPC–SPL) (*t*_173_ = 7.67, *P* < 10^−9^, *d* = 0.55, 95% CI = 0.39–0.71). These regions overlap with sites at which vicarious activations have previously been reported—phenomena whereby observing the sensations of others recruits areas that normally process one’s own sensations^1^ (Extended Data Fig. 4f–g).

Importantly, the additional somatotopic territory revealed by video watching encompassed even classically defined ‘visual’ regions (Methods), which show increased somatotopic connectivity relative to rest (main effect of task: *F*_1,173_ = 132.26, *P* < 10^−22^) (Fig. 2c). Dorsal and lateral visual regions specifically showed the strongest increases (*P* < 10^−23^), consistent with the proposed roles of ‘vision for action’ and the dynamic aspects of social perception^24^, respectively (interaction between stream and task: *F*_1.71,296.48_ = 27.73, *P* < 10^−9^).

## Multimodal connectivity in visual cortex

Our analyses of video watching reveal somatotopically structured activations in extrastriate cortex, prompting the question of how they relate to retinotopic activations more traditionally associated with this region. Our simultaneous estimation of somatosensory and visual connective fields, with automatic variance partitioning (Extended Data Fig. 1a–f), enables quantification of the relative importance of somatotopic and retinotopic activations in explaining BOLD responses, thereby testing the centrality of somatotopic responses in visual cortex.

**Fig. 3:**
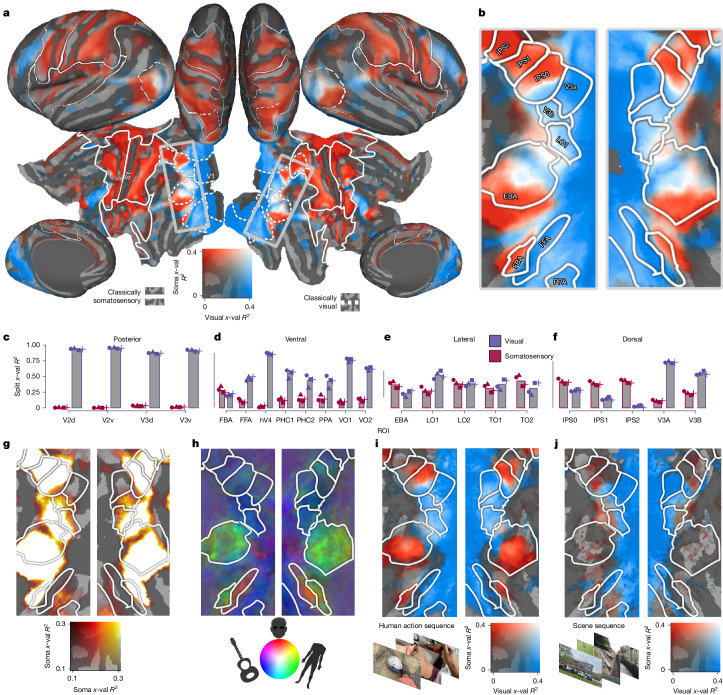
Multimodal topographic connectivity in dorsolateral visual cortex. **a**, Connective-field model performance from each source region (V1 (blue) and S1 (red)), quantified by cross-validated explained variance. The solid and dashed overlays indicate the somatotopic ROIs and conventionally defined visual regions shown in Fig. 2. The rectangle marks a band of dorsolateral visual cortex shown in the following panels. **b**, Enlarged portion of extrastriate cortex in which preferred connective-field modality reveals bilaterally symmetric alternations. **c**–**f**, Region-based (occipital (**c**), ventral (**d**), lateral (**e**) and dorsal (**f**)) average of S1- and V1-connective-field-derived cross-validated variance explained. In this average-participant representation, several regions in traditionally defined visual cortex (FBA, EBA, TO2 and IPS0–2) show a somatosensory-dominant connectivity profile. The symbols indicate estimates from video and participant half-splits of the data. *n* = 4 folds, derived from *n* = 174 participants for video folds and *n* = 87 for participant folds. *x*-val *R*^2^, cross-validated variance explained. **g**, Unimodal (somatotopic) cross-validated variance explained. Variance explained drops off ventrally to the FBA and posteriorly to LO1/V3B, where retinotopic responses dominate; these regions were therefore not considered to be valid somatotopic regions for further analysis. **h**, The RGB colour map depicts visual selectivity for faces, bodies and objects (peak-normalized *t*-statistics) from an independent functional localizer. The EBA and FBA appear as two green regions in lateral and ventral visual cortex, overlapping areas with dominant somatotopic connectivity apparent in **b**. **i**,**j**, The same outcomes as in **b** are shown, estimated from fitting to a video sequence involving human actions (**i**) and fitting to a video sequence depicting a movement through scenes, with no humans present (**j**). The images in **i** and **j** are original photographs taken by the authors, but are composed to be representative of those contained in the relevant HCP video sequences.

Figure 3a–f shows the balance of explained variance by visual (blue) and somatosensory (red) connective fields in the average participant. Occipital visual cortex appears strongly blue and the endogenous somatotopic network is centred on the central sulcus and appears strongly red, indicating unimodal activation. Between these regions, dorsolateral visual cortex exhibits a bilaterally symmetric band of alternating somatotopic and retinotopic connectivity strength (Fig. 3b).

**Extended Data Fig. 6:**
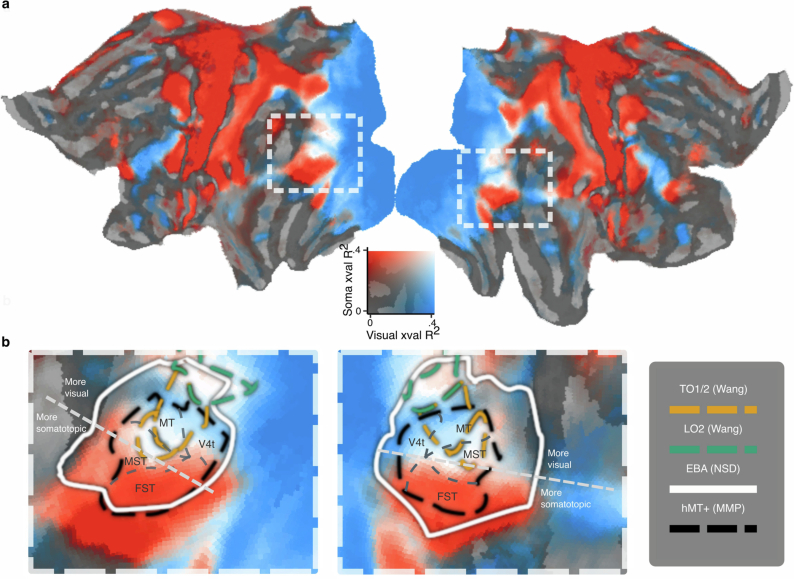
Organization of EBA and overlapping regions in relation to multimodal topographic connectivity. **a**. Connective field strength from each of the source regions (V1 - blue, S1 - red) is quantified by their separate cross-validated variance explained. The dashed rectangle depicts an area of lateral visual cortex that encompasses the EBA. **b**. Cutout depicts an enlarged version of the data within the rectangle defined in **a**. The legend depicts ROI definitions for EBA, retinotopic regions LO2, TO1-2 and the human MT+ complex, for which subfields (MT, V4t, MST, FST) are labelled on the flatmap. An additional dotted white line is drawn to demarcate a modality shift from retinotopy dominance to somatotopy dominance, which aligns well with the border of retinotopic region TO2. More posterior hMT+ fields (MT, V4t) show a retinotopy dominance and the more anterior fields show a somatotopy dominance (MST, FST).

Although far-flung from classical somatosensory regions, much of this region shown in Fig. 3b exhibits multimodal topographic connectivity (that is, retinotopic and somatotopic) during video watching. Taking the extrastriate body area (EBA) as an example, our model reveals alternating modality preferences that clarify its diverse functional attributions^25^. Specifically, the more posterior–dorsal portion, which is primarily retinotopic (blue), overlaps with temporal-occipital (TO1, TO2) and lateral occipital (LO2) retinotopic regions and terminates at their anterior boundary^26^. By contrast, the more anterior–ventral portion is more somatotopic (red), overlapping with the anterior fields of the motion-sensitive MT+ complex^18^ (Extended Data Fig. 6) and extending anteriorly beyond the EBA, consistent with the proposed action-related region partially overlapping with the EBA^27^. We note that the EBA is one of several dorsolateral visual regions of which the signal variance is best explained by topographic connectivity with S1 rather than V1 (Fig. 3b–f).

**Extended Data Fig. 7:**
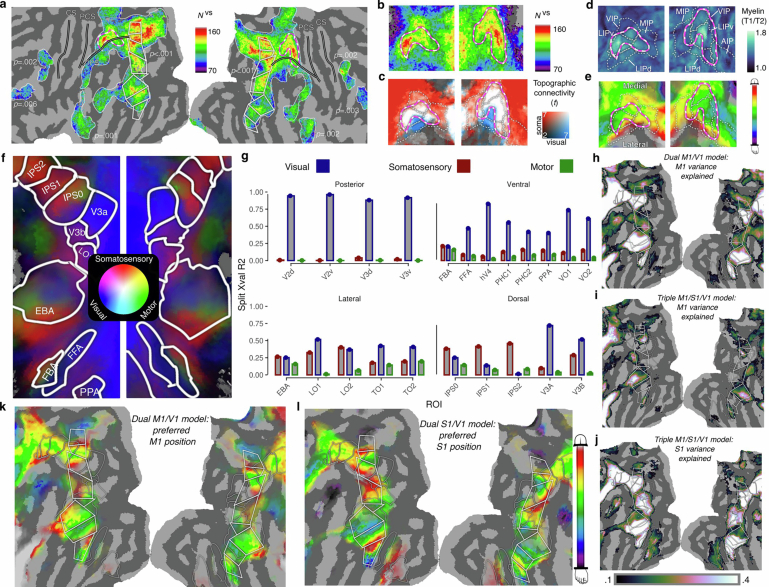
Individual subject reliability of multimodal topographic connectivity and M1 analysis outcomes. **a**. For each vertex location, the colour depicts the number of subjects with both above threshold visual and somatosensory model performance (*N*^*vs*^). The data are masked to show clusters of *N*^*vs*^ detected by permutation analysis (see Methods). The pink overlay highlights a posterior parietal region of reliably above-threshold multimodal connectivity near to the confluence of the postcentral sulcus (PCS) and intraparietal sulcus (IPS). **b**. Shows zoomed in view of the data in **a** centred around this posterior parietal region. **c**. 2D colormap depicts group-level *t* statistics for topographic connectivity in the somatosensory (red) and visual (blue) modalities. Much of this region appears white, indicating high levels of topographic connectivity in both modalities. **d**. Depicts myelin density (T1/ T2). Overlays depict overlapping regions of interest (see Methods). The region of reliable multimodal connectivity overlaps with an isolated ‘hotspot’ of high myelin density in LIP. **e**. Colours depict preferred S1 position. Connectivity to the face region of S1 is represented more laterally. **f**. RGB colormap depicts the cross-validated split variance explained by triple-source connective field modelling from S1 (somatosensory/red), M1 (motor/green) and V1 (visual/blue). **g**. Bar charts depict visual cortical region-based average of S1, M1 and V1-derived cross-validated variance explained for the respective connective field model. In all regions of visual cortex, responses are better explained by connective fields deriving from S1 than from M1. Data are derived from the ‘average subject’ (see Methods, derived from *N* = 174 subjects). **h**. Depicts cross-validated variance explained for M1 connective fields within the context of a dual model in which they compete alone for variance explained with V1. **i**. Depicts the same outcomes, derived from the triple-source model in which M1 connective fields compete for variance explained with both S1 and V1. **j**. Depicts cross-validated variance explained for S1 connective fields in the context of the same triple source model. Although M1 connective field models perform well when competing with V1 (**h**), S1 connective fields are far superior to M1 in explaining dorsolateral visual cortex responses when all source regions compete (**i-j**). **k-l**. Depicts preferred position on M1 and S1 according to the dual source models. A map-like arrangement with reversals in similar locations can be observed for connectivity to both M1 and S1 source regions.

Across individuals, all lateral and dorsal ROIs of the visual system (except for V3A) exhibited multimodal topographic connectivity (maximum somatotopic *P* = 0.024, maximum retinotopic *P* < 10^−22^). In ventral regions, this multimodal connectivity was unique to the fusiform body area (FBA) (retinotopic *P* < 10^−22^, somatotopic *P* < 10^−8^), sharply distinguishing it from the neighbouring fusiform face area (FFA) (Fig. 3b) and more ventral retinotopic regions (ventral-occipital areas (VO1–2), parahippocampal areas (PHC1–2)), which show only retinotopic activations (Fig. 3d). Individual-level analyses confirmed consistent loci of multimodal connectivity across individuals, concentrated in dorsolateral visual cortex and an additional ‘hotspot’ in posterior parietal cortex (Methods and Extended Data Fig. 7), potentially corresponding to a visuotactile map reported previously^28^. These findings establish that somatotopic processing may be unexpectedly pivotal in responses to naturalistic visual stimulation in the lateral and dorsal, but not ventral visual system.

We next examined what cognitive aspects driven by video watching drive these somatotopic responses in visual cortex. Importantly, while video watching introduces exogenous stimulation relative to resting-state, it does so in the visual domain—a visually driven recruitment of the sensorimotor system resembling mirror neuron activations^29^. This interpretation predicts a predominant role of motor cortex rather than somatosensory cortex^1^. Using M1 as an additional, third source region we found that S1 connective fields greatly outperformed M1 connective fields in explaining dorsolateral responses during video watching (Extended Data Fig. 7). This superiority points to the primacy of visually driven somatosensation over simulated or executed motor programs in explaining signal variance^29^, speaking against a strict mirror-neuron-like interpretation of our results.

One distinct possibility is that somatotopic responses in dorsolateral visual cortex reflect vicarious activations to video content. This generates two predictions: first, that such responses would be greater in regions sensitive to visual presentations of human agents; and, second, that they are modulated by visual content, such that they increase when human agents are presented. Indeed, a notable feature of the data in Fig. 3a,b is that the location of predominant somatotopic responses overlaps substantially with known locations of regions with robust preferences for visual presentations of bodies: the EBA and FBA^30^. Referencing against independent visual functional localizer data (Fig. 3h) confirmed that somatotopic responses associated more with body than face (*P* < 10^−4^), object (*P* = 0.012) or place (*P* = 0.028) selectivity, indicating visual-category-selective somatotopic responses. Testing this content dependence, we repeated our analysis on two video segments: one that depicts human agents interacting with objects and one in which the camera pans through various environmental locations with no human agents present. This revealed that the somatotopic responses in the EBA (*t*_173_ = 3.70, *P* < 10^−3^, *d* = 0.28, 0.13–0.43) and FBA (*t*_173_ = 3.06, *P* = 0.001, *d* = 0.23, 0.08–0.38) were indeed contingent on viewing human agents, with both regions exhibiting greater somatotopic connectivity during the human sequence (Fig. 3i,j). These results suggest that these somatotopic responses do not reflect spurious factors such as generic responses to visual input or bodily engagement with camera movements. Furthermore, the association between such visual-content-driven responses and regions of established selectivity to viewed human agents is consistent with vicarious responses^1^.

## Somatotopic maps tile visual cortex

**Fig. 4:**
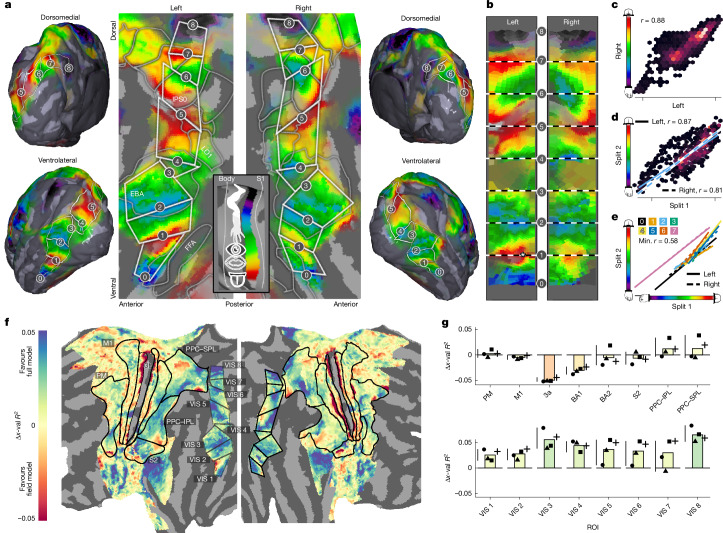
Structured somatotopic representations in dorsolateral visual cortex. **a**, The preferred S1 position estimated by the connective-field model. Reversals are demarcated by white polygons. No evidence for somatotopic connectivity is detected in regions appearing below (ventral) to FBA, and these areas were not further analysed. Data are thresholded to show vertices with out-of-set variance explained > 10% and transparency weighted between 10 and 40%. **b**, Voronoi plot of the data in **a**. Projective transformation was used to render the polygons in **a** into rectangles and concatenated them along the principal axis of the gradient, thereby rendering each hemisphere’s maps into common coordinates. The asterisk demarcates a strong left-hemisphere face representation that overlaps with the VWFA. **c**, Hexbin plot showing the agreement between data in the left and right hemisphere. This correlation was carried out on the data rendered into the same space as **b**, with the right hemisphere flipped about the *x* axis to point in the same anterior–posterior direction as the left hemisphere. **d**, Hexbin plot showing the agreement between independent participant splits; the least-squares fit to the data is shown for each hemisphere. **e**, Least-squares fits for the relationship between the preferred S1 position in each of the 16 maps across independent participant splits. The map is indicated by the colour and the hemisphere is indicated by the line type. **f**, The difference in cross-validated variance explained (Δ*x*-val* R*^2^) between the best body part field model (Methods and Extended Data Fig. 8) and the full model (blue, favours full model; red, favours field model). VIS denotes that the region resides in visual cortex. **g**, The ROI-based average Δ*x*-val* R*^2^. The symbols indicate estimates from video and participant splits of the data. *n* = 4 folds, derived from *n* of 174 participants for video folds and *n* = 87 for participant folds. 3a, Brodmann area 3a; PM, premotor cortex.

**Extended Data Fig. 8:**
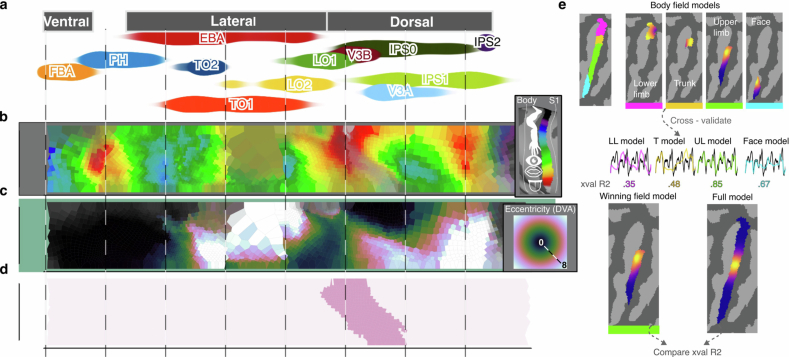
Additional searchlight analyses and alternative field-based connective field model. **a**. Density plots depict the location of ROIs along the ordinate (roughly ventral-dorsal) of the rectangular space defined in Fig. 4b. **b**. Voronoi plot of the same somatotopic map depicted in Fig. 4b. **c**. Depicts visual field eccentricity (DVA) as determined from the HCP retinotopy dataset. **d**. Depicts the geodesic chunks wherein evidence of alignment between eccentricity and the somatotopic map was detected. **e**. Depicts the model-fitting procedure for the alternative field-based models. Four additional connective field models are fitted using restricted source regions of the 3b subsurface, corresponding to the lower limb, trunk, upper limb and face fields (see Methods). The cross-validated variance explained of the winning field model is compared with the full model to compute a *∆xvalR*^2^ for model comparison.

Somatotopic connectivity explains substantial BOLD signal variance in dorsolateral visual cortex during video watching. This raises the possibility that, as in the core somatosensory network across frontal and parietal lobes, visual cortex body-part tuning may be structured as orderly, somatotopic map-like arrangements on the cortical surface. To test this possibility, we projected connective-field estimated somatotopic tuning onto this region. In the HCP-average participant, this revealed multiple gradients of connectivity-derived body-part tuning separated by reversals, one canonical signature of the presence of cortical field maps (Fig. 4a). This somatotopic structure, shown in Fig. 4b, was highly consistent between hemispheres both across maps (*r* = 0.88, *P* < 10^−16^; Fig. 4c) and on an individual-map-level basis (Fig. 4e). Although lower limb representation in this region appears sparse, the size of connective fields is large, with 16% of the connectivity deriving from the lower limb field. Indeed, we fit an alternative connective-field model (Methods and Extended Data Fig. 8e) constraining connective fields within discrete body-part fields of S1. We find that there is increased evidence for this restricted model in patches of Brodmann area 3a, M1, premotor cortex, Brodmann areas 1 and 2, as well as in PPC–SPL and at the border between the SII and PPC–IPL (Fig. 4f), consistent with a field-based topographic organization that has been previously reported^17^. By contrast, the full model performed better in caudal PPC and in dorsolateral visual cortex (Fig. 4g). These findings suggest a hybrid organization—discrete field-based topography in core somatosensory regions and integrative cross-field tuning in higher-order parietal and visual areas.

**Extended Data Fig. 9:**
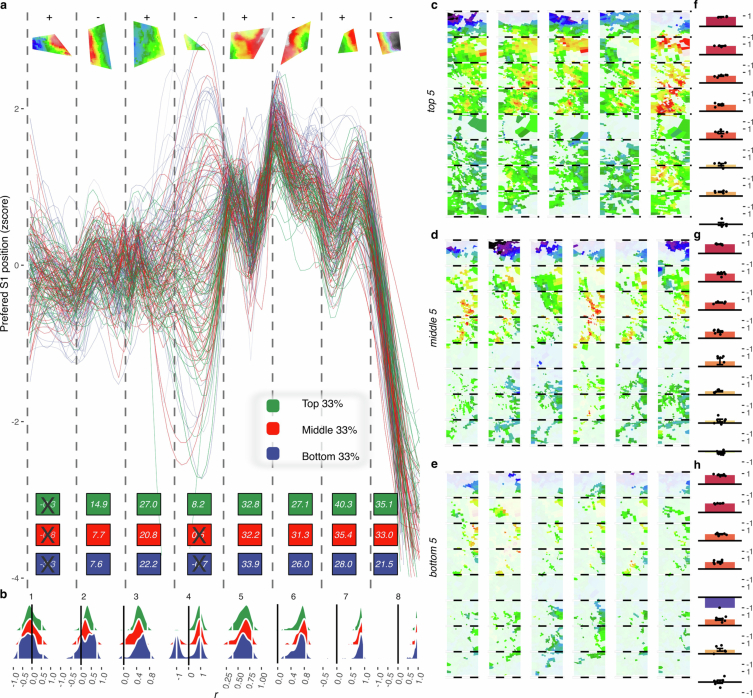
Individual subject variability in dorsolateral somatotopy. **a**. Lines depict preferred S1 position estimated by the connective field model, as a function of the principal axis of the somatotopic reversals depicted in Fig. 4. Colour depicts the data from the top, middle and bottom third of subjects according to cross-validated variance explained in this region. The numbers represent the signed log10 transformed *p* values for a one sample *t*-test of the Fisher’s *z* correlations with the average-subject data against zero for the groups defined above (colours). To visualize relative anatomical scale, the polygons at the top of the plot depict the data from the average subject outcomes reported in the main text. Outcomes deriving from smaller regions are generally more variable (map 1, map 4), likely because of concomitant influence of anatomical variability and thresholding having more deleterious impact in regions with fewer vertices. **b**. Depicts the distribution of individual subject correlations (Pearson’s *r*) with the average-subject data as a function of map. **c-e**. Individual subject maps for the top, middle and bottom 5 subjects, according to cross-validated variance explained. **f-h**. Mean correlation with the average-subject data as a function of map and subject group. Dots represent the correlations for the individual subjects depicted in **c** (*N* = 5). All data on this plot are thresholded according to procedures described in the Methods.

To test the generalization performance of our estimates of somatotopic structure in this region, we repeated our model fitting on a random half of the participants (*n* = 87) and correlated the resulting somatotopic map with the remaining independent half (*n* = 87). The map predicted out-of-set data well (*r* = 0.84,* P* < 10^−16^) (Fig. 4d). Robustness was further tested with a conservative permutation test that generates alternative surrogate somatotopic maps maintaining autocorrelation of the empirical data (Methods). This revealed that such out-of-sample agreement of this magnitude was rare with surrogate instances (*P* = 0.026). Individual-participant variability is summarized in Extended Data Fig. 9. Although substantial variability can be observed across individual data, 7 out of 8 maps shown in Fig. 4a robustly correlated with individual-participant estimates, highlighting the generalizability of this organization.

Notably, we observed the strongest hemispheric asymmetry in the most ventral map (maximum *P* < 10^−3^ for pairwise comparisons of bootstrapped absolute hemispheric differences), driven by stronger upper-limb/face representation in the left hemisphere (Fig. 4b (asterisk)). This map, spanning the region between the EBA and FBA, overlapped the visual word form area (VWFA)^31^ —a left-lateralized region functionally linked to S1 in both people with limited and full vision during manual exploration of tactile patterns^32^.

## Body-part tuning predicts visual function

**Fig. 5:**
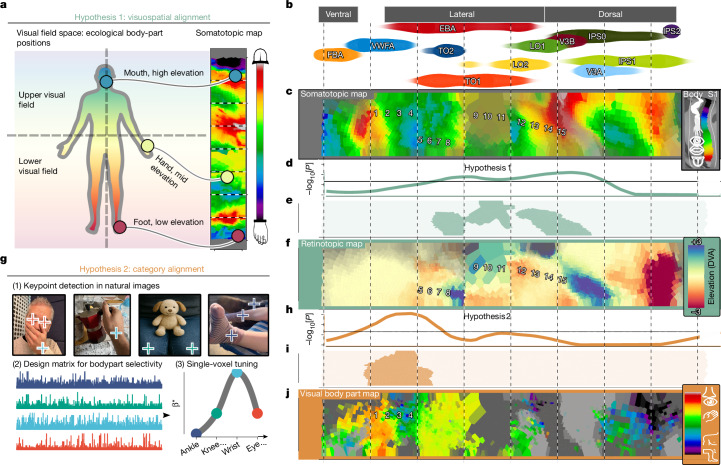
Reference frames of processing predicted from somatotopic connectivity. **a**, Schematic of the topographic alignment predicted by visuospatial alignment: voxels tuned to upper visual field locations are expected to coincide with parts of the somatotopic map corresponding to high-elevation body parts (and vice versa). **b**, The location of ROIs along the ordinate (roughly ventral–dorsal) of the rectangular space defined in Fig. 4b. **c**, Voronoi plot of the same somatotopic map depicted in Fig. 4b. Numbers are overlaid to aid visual comparison of corresponding points in ensuing figures. **d**, Evidence for alignment between the somatotopic map and retinotopic map shown in **f**, defined as maximum −log_10_[*P*] of the chunk-wise correlation. **e**, The geodesic chunks in which evidence of alignment was detected. **f**, The vertical visual field position (*y*) as determined from the HCP retinotopy dataset. **g**, The procedure for deriving a map of visual body-part selectivity. For NSD images, 17 anatomical keypoints were detected (4 are shown to prevent overplotting) to compute predictions that model selectivity for each body part (Methods). Images are original photographs taken by the authors, but are composed to be representative of the COCO images predicted to elicit the peak response for an eye-, wrist-, knee- and ankle-selective voxel, respectively. **h**, The evidence for alignment between the somatotopic map and visual body part map shown in **j** defined as the maximum −log_10_[*P*] value (average participant permutation test outcomes, derived from *n* = 174). **i**, The geodesic chunks for which evidence of alignment was detected. **j**, Visual body part preference data generated from the procedure shown in **g**. The colour bar shows the position of bodily keypoints along this axis. All data in the right column are nearest-neighbour averaged across hemispheres. Searchlight analyses were performed separately on each hemisphere before averaging of outcomes was applied.

Having established somatotopic organization in dorsolateral visual cortex, we next examined how the computational machineries of vision and somatosensation might be configured to connect to and recruit one another in this region. As much of the visual system is retinotopically organized, one elementary way in which this may be implemented is through alignment of the somatotopic and retinotopic reference frames. Such alignment could reflect ecological coincidences between visual field and bodily positions (for example, feet in the lower visual field) supporting computation of environmental affordances (hypothesis 1; Fig. 5a). To test hypothesis 1, we performed a permutation-based searchlight analysis to detect agreements between the somatotopic map and preferred vertical visual field position estimated from HCP retinotopy data (Fig. 5f and Methods). We detected evidence for this alignment mostly in dorsal regions spanning retinotopic region V3B (*P* < 10^−2.12^) and laterally in LO1 (*P* < 10^−2.12^) and superior EBA (*P* < 10^−1.90^), consistent with the robust connectivity of the EBA with the dorsal visuomotor stream^33^ and its role in action-related processing^25^ (Fig. 5b–f).

Ventrally, in the region spanning superior FBA, inferior EBA and the VWFA, we detected no evidence for the above-described alignment between somatotopic and retinotopic tuning, probably reflecting the strong foveal bias of this region. While the VWFA is not classically defined as body selective to the same extent as the FBA and EBA, its activity has been linked to non-textual gestures of the hands and face^34^. We therefore reasoned that topographic alignment here may occur at a categorical level, such that somatotopic structure predicts visual body-part selectivity. If this were the case, we should be able to predict voxel-level visual selectivity to body parts from our somatotopic map (hypothesis 2; Fig. 5g).

To test hypothesis 2, we used the Natural Scenes Dataset (NSD)—a 7 T functional magnetic resonance imaging (fMRI) study in which observers viewed 10,000 natural images^35^. We used a pose-estimation algorithm to detect 17 keypoints within each of the viewed images, ranging from lower body parts (such as the ankle) to upper facial features (for example, the eyes). This visual annotation formed a design matrix for a forward model of visual selectivity, from which single-voxel visual body part tuning could be derived (Fig. 5g and Methods) to estimate preferred visual body position along a toe–tongue axis analogous to the somatotopic map (Fig. 5j). The searchlight analysis revealed that the somatotopic map predicted visual body part preference from the superior portion of FBA into the ventral portion of the EBA (*P* < 10^−2.94^) and VWFA (*P* < 10^−2.94^) (Fig. 5h–j). These findings suggest at least two complementary multisensory alignment modes in visual cortex: visuospatial dorsally and semantic ventrally, converging laterally—consistent with lateral visual cortex’s role as a bridge between the dorsal action and ventral perception streams^33^.

## Discussion

A central question in sensory neuroscience is how inputs from vision and touch are combined to generate cohesive representations of the external world. Here we reveal a widespread mode of brain organization in which aligned topographic maps bridge vision and somatosensation. We developed a computational model that revealed somatotopic structure in dorsolateral visual cortex. Somatotopic tuning in these regions was predictive of visual field locations more dorsally and visual body part selectivity more ventrally. These results suggest more extensive cross-modal overlap than traditionally assumed: the computational machinery classically attributed to the somatosensory system is also embedded within and aligned with that of the visual system. These aligned visual and bodily maps are a likely brain substrate for internalized somatosensory representations of visual signals, and are a candidate human homologue of findings in mice whereby somatomotor responses dominate visual cortex^36^.

Consistent with embodied perception theories, our model-based quantifications of somatotopic and retinotopic connectivity revealed that dorsolateral visual cortical responses to naturalistic stimuli are best explained by selectivities in both modalities, as opposed to visual selectivity alone. The necessity of incorporating body-referenced processing into models of dorsolateral visual cortex supports evidence that its role extends beyond passive visual analysis, encompassing perceptual, semantic and bodily functions optimized for behavioural interactions with the world^25^.

Consistent with visuospatial alignment of somatosensory tuning, we found that body part preferences in dorsolateral visual cortex predicted visual field tuning. Such alignment, previously reported at the terminus of the dorsal visual pathway around the postcentral sulcus^28^, therefore extends far into dorsal and lateral streams of the visual system. This alignment may be reinforced by shared developmental influences, as somatotopic and retinotopic maps are shaped trophically from birth: dorsal regions represent the upper body and visual field, and ventral regions to the lower body and visual field^22^, providing a roughly aligned sensory periphery optimized for efficient environmental sampling and action. The explicit interweaving of touch and retinal coordinates may subserve efficient perception of environmental affordances and a cohesive sense of spatial self-representation. Conversely, the imposition of a visuospatial reference frame on somatosensation may contribute to the visuospatially dependent ‘rubber hand illusion’ of spurious limb ownership^37^, and to reduction of touch sensitivity in crossed arms, which is absent in the congenitally blind^38^.

**Extended Data Fig. 10:**
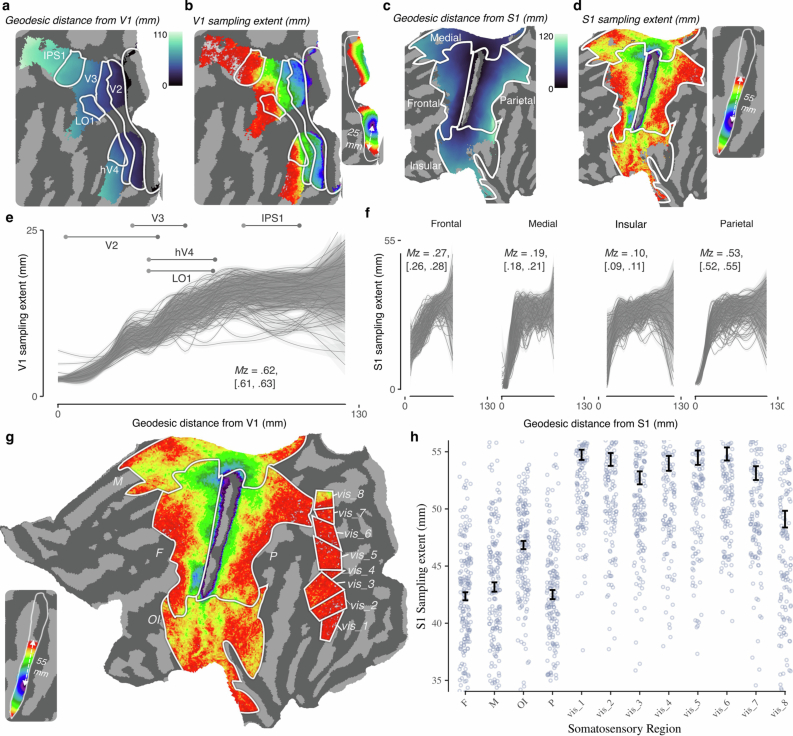
Quantifications of connective field sampling extent. **a**. The colours depict geodesic distance from V1 defined in mm. **b**. Depicts the average V1 sampling extent of connective fields in mm. The panel to the right provides a reference scale for the colormap on the V1 surface. **c-d**. The same as **a-b** are shown for S1 connective fields. **e**. Depicts V1 sampling extent as a function of geodesic distance from V1. Lines are individual subject data. For reference, the labelled horizontal lines depict the locations of a subset of visual regions that are depicted on **a-b**. **f**. The panels show S1 sampling extent as a function of distance from S1 for 4 gross-anatomical somatosensory regions whose locations are drawn on **c-d**. **g**. Depicts S1 sampling extent for 4 gross-anatomical somatosensory regions as well as 8 dorsolateral visual maps. **h**. S1 sampling extent is shown as a function of the 12 regions depicted in **g**. Error bars are *M* ± 1SE. Points reflect individual observers (*N* = 174).

Aspects of this mapping may adapt. A body-referenced computational machinery shared between the visual and somatosensory systems may support cross-modal plasticity: regions of dorsolateral visual cortex can support tactile discrimination tasks (such as Braille reading) after less than a year of training^32^. In addition to being prone to reorganization, the structured map-like organization betrays these regions’ flexibility. An analysis of connective-field sampling extent in dorsolateral visual cortex indicates sampling of large portions of the somatosensory body map, comparable to that observed in posterior parietal regions of somatosensory association cortex (for example, Brodmann areas 5 and 7; Extended Data Fig. 10). There, single-cell recordings indicate receptive fields spanning multiple body parts^39^. Moreover, high-level visual cortical responses are characterized by substantial invariance to position and extent of stimuli, aiding processes such as visual object recognition^40^. This invariance principle may extend to visuotactile integration, enabling flexible registration of common causes of visuotactile inputs despite variable body part positions (for example, hands being displaced in the visual field due to their variable ecological positions). Extensions of our topographic connectivity framework may help to chart these flexible nonlinear interactions.

We also found that somatotopic connectivity in a region overlapping with the VWFA predicted a map of visual body part selectivity mapped without reference to visual location. This congruence may support behavioural cross-modal modulations: observing a body part can improve tactile localization on it^41^, and visual judgements of body parts are enhanced when moving the corresponding body part^42^. For such cross-modal modulations to aid imitative visuomotor behaviours, they would require categorical visual representations of body parts robust to changes in size or viewpoint. This tuning could allow body-referenced computations to scaffold semantic cognition.

The convergence of somatosensory and visually referenced body part maps overlapped with the EBA, implicating this region in connecting felt and observed bodily sensations. This is consistent with causal evidence: electrical stimulation of the EBA can lead to judgements of one’s own bodily sensations being biased towards observed ones^43^. We also observed this body-referenced tuning alignment in the VWFA—a region that is responsive to both visual word forms but also by communicative hand and face gestures^34^. Such properties suggest that it functions as a likely conduit between visual, body-related and more-abstract semantic processes relevant to language. Electrical stimulation of sensorimotor regions supporting specific body movements (such as the leg) can influence the processing of words semantically related to associated actions (for example, kick). This supports models of embodied language, which propose that lexical processing is supported by functional motor language links. Our findings highlight that this principle can be extended to additionally incorporate similar contributions from vision^44^.

Interpreting our findings requires acknowledging fundamental disjunctures between vision and somatosensation. In vision, neighbouring V1 locations map to adjacent positions in sensor space, whereas, in S1, adjacent cortical representations (such as hand and face) correspond to non-contiguous body parts. Although Penfield’s classic homunculus depicts S1 as a continuous toe-to-tongue map, recent evidence suggests finer subdivisions into body part fields defined by myelin and connectivity^17^. Indeed, we found that connective-field models constrained by body field boundaries outperform a continuous connective-field model in much of classically defined somatotopic cortex (Fig. 4f,g). Yet somatosensory representations even in S1 are also found to be more distributed than the classical map suggests—for example, hand-related activity can be decoded from the functionally defined ‘foot’ region^45^. This distributed coding is consistent with proposals that sensorimotor cortex integrates body part information for action planning^46^. Collectively, these findings highlight multiple, still unresolved, somatosensory encoding schemes that challenge traditional cortical mapping^47^.

Our findings also underscore the unique potential of very large ultra-high-field MRI datasets—both in terms of width (that is, numbers of participants) and depth (that is, volume of data per participant)—as well as the potential of naturalistic stimuli in revealing previously undetected features of brain organization. Naturalistic stimulation generally evokes stronger, more specific activity than artificial stimuli, reflecting the brain’s optimization toward deriving meaning from fast, multisensory input in real-world contexts^48^. Topographic connectivity offers a means to track cross-sensory fusion both in controlled tasks and in naturalistic contexts that confound traditional approaches. We show that detailed sensory tuning can be inferred from BOLD responses during rest, and even more robustly during video watching. This holds promise for studying conditions such as autism, which are often characterized by visual and tactile disturbances that arise early in development^49^. Conventional mapping with sparse, repetitive stimuli is often impractical in young populations with sensory hypersensitivities and comorbid epilepsy^50^. By contrast, our task-free, stimulus-independent approach may broaden the viable playing field for studying sensory dysfunction.

Ultimately, these findings reveal a fundamental principle of brain organization, whereby alignment of visual and somatosensory maps may serve as a mechanistic substrate for our embodied visual experience of the world. This computational motif is a prime candidate for the bridge between perceptual and higher cognitive processes in the human brain.

## Methods

### Participants and stimuli

fMRI data were taken from 174 participants of the HCP movie-watching dataset^51^. The sample consisted of 104 female and 70 male individuals (mean age 29.3 years, s.d. = 3.3) born in Missouri, USA. In total, 88.5% of the sample identified as ‘white’ (4.0% Asian, Hawaiian or Other Pacific Island; 6.3% Black or African American; 1.1% unreported). The English language comprehension ability of the sample (as assessed by age-adjusted NIH Picture Vocabulary Test^52^ scores) was above the national average of 100 (mean = 110, s.d. = 15). The participants were scanned while watching short (ranging from 1 to 4.3 min in length) independent and Hollywood film clips that were concatenated into four videos of 11.9–13.7 min total length. Before each clip, and after the final clip was displayed, there were 20 s periods in which there was no auditory stimulation and only the word ‘REST’ presented on the screen. There were four separate functional runs, in which observers viewed each of the four separate videos. All four videos contained an identical 83 s ‘validation’ sequence at the end of the video that was later removed to ensure independent stimulation in each cross-validation fold. Audio was scaled to ensure that no video clips were too loud or quiet across sessions and was delivered by Sensimetric earbuds that provide high-quality acoustic stimulus delivery while attenuating scanner noise. The participants also took part in one hour of resting state scans, also split into four runs of equal (around 15 min) length. Full details of the procedure and the experimental setup are reported in the HCP S12000 release reference manual^53^. The ethical aspects of the HCP procedures were approved by Washington University Institutional Review Board (IRB) (approval number 201204036) and all use of the data reported in this manuscript abide by the WU-Minn HCP Consortium data use terms.

### HCP data format and preparation

Ultra-high field fMRI (7 T) data from the 174 participants were used, sampled at 1.6 mm isotropic resolution and a rate of 1 Hz (ref. ^51^). Data were preprocessed identically for video watching and resting state scans. For all analyses, the FIX-independent-component-analysis-denoised time-course data, sampled to the 59,000 vertex-per-hemisphere through the areal feature-based cross-participant alignment method (MSMAll)^54^ surface format was used. These data are freely available from the HCP project website. The MSMAII method is optimized for aligning primary sensory cortices based on variations in myelin density and resting state connectivity maps^18^. Owing to the unreliable relation between cortical folding patterns and functional boundaries, MSM method takes into account underlying cortical microarchitecture, such as myelin, which is known to match sensory brain function better than cortical folding patterns alone^55^. Previous research has demonstrated that such an approach improves the cross-participant alignment of independent task fMRI datasets while at the same time decreasing the alignment of cortical folding patterns that do not correlate with cortical areal locations^54^.

We applied a high-pass filter to the timeseries data through a Savitzky Golay filter (third order, 210 s in length), which is a robust, flexible filter that allowed us to tailor our parameters to reduce the influence of low frequency components of the signal unrelated to the content of the experimental stimulation (for example, drift, generic changes in basal metabolism). For each run, BOLD time-series data were then converted to percentage signal change.

For purposes of cross-validation, we made training and test datasets from the full dataset. We removed the final 103 s of each functional run, which corresponded to the identical ‘validation’ sequence and final rest period at the end of each video run. Our training dataset therefore consisted of the concatenated data from the four functional runs with this final 103 s removed from each. The test dataset was created by concatenating the final 103 s from each run into a 412 s set of data.

All connective-field models were fit on the individual-participant data and for video watching these models were also fit to the data of an across time-course averaged (HCP average) participant. Split-half participant averages (*n* = 87) were also created through a random 50% split of individual-participant data. Split-half video averages were created by creating separate datasets based on the first (videos 1 and 2) and second half (videos 3 and 4) of the videos.

### Dual-source connective-field model

#### Model maps of V1 and S1 topography

Our analyses extend the approach of connective-field modelling, wherein responses throughout the brain are modelled as deriving from a ‘field’ of activity on the surface of a ‘source’ region—classically V1. In turn, preferences for positions on the visual field can be estimated by referencing the estimated connective-field V1 positions against the retinotopic map of V1 (Fig. 1a–c). Here we extend this approach by simultaneously modelling brain responses as deriving from connective fields on both the V1 and S1 surfaces. This requires defining both a V1 and S1 source region and their underlying topographic maps.

To define these V1 and S1 source regions, we defined subsurfaces (one for each hemisphere) from the full cortical mesh, containing the vertices of a multimodal parcellation of the HCP data for regions V1 and Brodmann area 3b^18^. We chose region 3b as it is the first cortical input stage for tactile processing and is the most topographically organized subregion of S1.

To provide a model retinotopic map for V1, we used data from a ‘retinotopic prior’ that defines participant-averaged parameters of preferred visual field position (eccentricity, polar angle) estimated from a population receptive field (pRF) analysis of the HCP data^56^. Thus every vertex in V1 was associated with an eccentricity and polar angle value that defined its preference for the corresponding position in the visual field. Using this data, the V1 subsurface was then curtailed to only include vertices within the region stimulated by the video display (within 8 degrees of visual angle (DVA) from the fovea).

The known topographic organization of S1 is a somatotopic map, which is an approximately dorsomedially to ventrolaterally oriented gradient that runs from sensitivity to lower limbs to the upper limbs and face^13^. To provide a continuous coordinate space for this somatotopic gradient, for each vertex, we calculated the geodesic distance from the vertices at the most dorsomedial edge of the 3b subsurface.

#### Design matrix

We first summarized the V1 and S1 subsurfaces as a finite set of spatial profiles by deriving eigenfunctions of their Laplace–Beltrami operator (LBOEs) using functions contained within Pycortex^57^. This decomposition, referred to as recovering the shape DNA of a manifold, yields a finite family of real-valued functions that are intrinsic to the surface shape, orthogonal and ordered according to spatial scale^58,59^ (Extended Data Fig. 1a). In principle, one can approximate any arbitrary spatial pattern on the surface (that is, a connective field) through a linear combination of LBOEs.

To validate this approach and determine the number of LBOEs to use in our analysis, we conducted pilot analyses where we attempted to predict target Gaussian connective fields of varying sizes from linear combinations of LBOEs. These analyses indicated that for both V1 and S1, 200 LBOEs were sufficient to adequately predict connective fields with a sampling extent of 2 mm, which approximates the lower bound of the known sampling extent of extrastriate cortex from V1 (that is, in V2)^14^. Reconstruction performance was at near-ceiling levels for sampling extents of 4 mm and above (Extended Data Figs. 2 and 3) and increasing the number of LBOEs from 200 led to trivial increases in reconstruction performance relative to the increases in computation time. Furthermore, visualizing the performance of a 200 LBOE model in predicting connective fields centred on each V1 and S1 vertex revealed no systematic spatial inhomogeneities (Extended Data Figs. 2 and 3). As such we opted to use 200 LBOEs per subsurface in our connective-field modelling.

With this approach validated, we generated model time courses for our design matrix via the dot product of the time-course data corresponding to each subsurface and each of the 200 corresponding LBOEs. Each model time course therefore reflects the sum of the timeseries data within the subsurface, weighted by one of the LBOEs (Extended Data Fig. 1b). The model time courses were then *z* scored over time and stacked to form a design matrix for model fitting. Thus, there were 800 regressors in our design matrix: 400 from V1 and 400 from S1 (200 per hemisphere), which were used to explain the BOLD responses during resting-state and video watching.

#### Model fitting

All model fitting was conducted in Python, exploiting the routines implemented by the ‘Himalaya’ package^60^. In ordinary least-squares (OLS) regression, one estimates weights *b*, such that data *y* are approximated by a linear combination of regressors *Xb* (Extended Data Fig. 1c). Here we used banded ridge regression, which belongs to a family of regularized regression techniques that estimate a regularization parameter *λ* to improve the generalization performance of OLS regression^61^. Banded ridge regression expands on these techniques by estimating a separate *λ* for separate feature spaces *i* of the design matrix *X*—thereby optimizing regularization strengths independently for each feature space (Extended Data Fig. 1d). Banded ridge regression therefore respects the fact that different feature spaces in the design matrix may differ in covariance structure, number of features and prediction performance—entailing different optimal regularization.

In the present case, our two feature spaces consisted of the visual and somatosensory modalities or, equivalently, the 400 V1 and S1 model time courses (X_*v*1_, X_*s*1_) described in the previous section. Thus, to model brain activity of a particular voxel, banded-ridge regression computes the weights b*_i_, as defined below:

$${b}^{* }=\mathop{{\rm{argmin}}}\limits_{b}{\Vert \sum _{i}{X}_{i}{b}_{i}-y\Vert }_{2}^{2}+\sum _{i}{\lambda }_{i}{\Vert {b}_{i}\Vert }_{2}^{2}.$$

Similarly to unbanded ridge regression, the ridge weights b*_i_ are estimated from the training data and the hyperparameters *λ*_*i*_ are learned through cross validation. In the present case, our training data consisted of four runs of functional data in which the participants watched an independent video. This natural organization of the data enabled us to use a leave-one-video-out cross-validation strategy to estimate *λ*_*i*_.

#### Connective-field estimation

For a given voxel, the coefficients b*_i_ estimated by the banded ridge regression model can be interpreted as the cross-validated importance of each model time course in the design matrix in explaining its response throughout the experiment (rest or video watching). By extension, as each model time course derives from an orthogonal spatial profile on the surface of V1 or S1, this means that b*_i_ also implicitly estimates the importance of each of the underlying spatial profiles. Accordingly, for any given voxel, the dot product of its estimated b*_i_ and the corresponding spatial profiles *s*_*i*_ reveals a spatial map of the importance of each vertex on S1 and V1 in explaining the voxels response—or equivalently—it estimates its visual and somatosensory ‘connective field’ (Extended Data Fig. 1e).

Notably, this method of connective-field estimation is more flexible than ‘classic’ connective-field estimation procedure as it removes the constraint that the connective-field profile is a Gaussian defined by a centre (*V*_0_) and extent (*σ*), and can estimate non-canonical or irregular spatial patterns that do not resemble unimodal and circularly symmetric Gaussians.

Furthermore, this form of connective-field modelling through banded ridge regression is highly extensible as it can incorporate additional source regions with different topographic formats (for example, primary motor cortex, primary auditory cortex) simply through expansion of the feature spaces and bands in the design matrix. Banded ridge regression is particularly suitable in this context of multiple, potentially correlated feature spaces, as the estimation of multiple regularization parameters also leads to implicit feature selection^60^. During cross-validation, banded ridge regression is able to learn to ignore some feature spaces to improve generalization performance. To ignore an uninformative feature space, banded ridge regression penalizes its influence by assigning a large regularization hyperparameter *λ*_*i*_, implying that the coefficients b*_i_ are shrunk toward zero. This process effectively removes uninformative feature spaces from the model.

We note that great care should be taken when interpreting connective-field modelling outcomes in regions directly abutting the source region. This is because results in these neighbouring regions are likely to be biased by partial-voluming effects: in this case, a connective field centred in the source region spuriously samples from the neighbouring target region itself across the border. This contamination can be due to several factors, among which the fact that single voxels can sample grey matter on both sides of the boundary on the surface (or across both banks of a sulcus), the broad scale of spatial autocorrelations of the underlying responses and the BOLD point-spread of around 2 mm, more than the voxel size in the acquisitions used here.

#### Connectivity-derived retinotopic and somatotopic mapping

With connective-field profiles estimated for each vertex, preferred visual field and body positions were estimated by taking the dot product of each S1 and V1 connective field and the corresponding somatotopic and retinotopic map (see the ‘Model maps of V1 and S1 topography’ section) and then dividing by the sum of the connective fields (Extended Data Fig. 1f). As the values of the connective-field profile represent the importance of each location on the source region in explaining a given voxels response, this is akin to a weighted averaging, whereby the retinotopic/somatotopic maps are averaged in a manner weighted by the predictive performance at each location.

To validate the ability of our model to simultaneously estimate retinotopic and somatotopic maps, we compared our connective-field-derived maps to independent maps derived from exogenous stimulation. For an exogenously derived retinotopic map, we leveraged the retinotopic prior previously described^56^. To obtain an exogenously derived somatotopic map, we used data from an independent, publicly available whole-brain somatotopy dataset collected at 3 T, where 62 participants performed movements with 12 discrete body parts ranging from toe to tongue^19,62^. The participant-wise *β* weights for each of 12 body parts were nearest-neighbour resampled into the same 59,000 vertex per hemisphere space as the HCP data. We then conducted a second-level GLM on these beta weights and then took the dot-product of the resulting group-average betas and their ordinal position on the S1 homunculus, resulting in a continuous toe–tongue metric of body part sensitivity. Note that, before the dot product operation, we averaged the leg *β* weight across left and right leg movements, as only this body part was represented bilaterally in the dataset.

Correlating our connectivity-derived topographic maps with these exogenous maps, we confirm that our outcomes recovered detailed somatotopic and retinotopic organization that closely mirrors those derived from exogenous stimulation. Notably, even in medial and insular regions, where the performance of the connective-field model is relatively low, many features of the exogenously derived somatotopic maps are clearly present in our own (Extended Data Fig. 5e–h).

#### Analysis of subfields within S1

To explicitly relate our connective-field coordinates to body parts, we leveraged the definitions of four topographic body part fields within our S1 source region (3b) provided previously^18^ (lower limb, trunk, upper limb and face), which were identified on the basis resting-state functional connectivity gradients and somatotopic mapping task contrasts. Note that these authors^18^ also report the existence of an additional eye field in some areas of sensorimotor cortex, but indicate that this is not reliably identifiable in area 3b; thus, this does not feature in our analysis. We corroborated the validity of these four subfields with data from the whole-brain somatotopy dataset described in the previous section. The second-level random effects GLM analysis was used to derive the S1 positions corresponding to the peak of the group-level *t* statistic for each body part. These data, alongside the topographic field boundaries, are shown in Extended Data Fig. 4d. All functionally defined peak statistics fell within the corresponding body part field defined in that study^18^.

With these fields defined, for each vertex, we summed the connective-field profile within each field and then normalized by the sum across all fields to estimate the proportion within each field. This measure therefore provides an estimate of the importance of each body part field in driving responses within the vertex, which we then compared between somatosensory regions of interest (Extended Data Fig. 4e).

#### Model performance metrics

The dual specification of our model with multiple feature spaces also enables us to disentangle the contribution of each feature space (sensory modality) to overall prediction performance. Specifically, variance decomposition through the product measure enables the computation of independent *R*^2^ scores per feature space that sum to the total *R*^2^ of the dual model.

$${\widetilde{R}}_{i}^{2}=\frac{\sum _{t}{\hat{y}}_{i}(2y-\hat{y})}{\sum _{t}{yy}}$$

Where ${\hat{y}}_{i}$ is the subprediction computed on feature space *X*_*i*_ alone, using the weights b*_i_ of the dual model. To evaluate out-of-sample performance of the model, the parameters estimated from the training data were then used to predict the test data and the variance explained from the V1 and S1 feature spaces was evaluated using the above formula.

### ROI definitions

#### Classically somatotopic ROIs

To define somatotopic regions of interest, we leveraged a previously defined, gross anatomical parcellation of parietal, medial, insular and frontal zones that have been found to contain robust homuncular somatotopic gradients, or ‘creatures’ of the somatosensory system^13^. These ‘creatures’ were themselves defined by a combination of regions in the multimodal parcellation of ref. ^18^, of which the voxel-averaged response to tactile stimulation was significantly above zero. Further details of the exact regions of the Glasser parcellation^18^ that correspond to each ROI can be found in another paper^13^. More granular regions of somatotopic cortex reported in the main text (3a, Brodmann area 1–2) were defined from individual Glasser atlas definitions. Other broader regions reported are combinations of multiple Glasser atlas regions: SII (OP1 and OP4) superior parietal lobule (7Am, 7PL, 7PC, 7AL, 7Pm, 7 M, VIP, MIP, LIPd, LIPv) inferior parietal lobule (PF, PFm, PFt, PGa, PGp PFop, PFcm).

#### Classically visual ROIs

To define visual ROIs, we used a pre-existing probabilistic atlas of 25 retinotopic visual regions provided in ref. ^26^. To this parcellation, we added the regions FFA, FBA, EBA and PPA, which were defined by functional localizer (floc) data taken from the NSD^35^. Specifically, the participant-averaged *t*-statistics for the faces/bodies versus all other stimulus categories contrast were thresholded at the *α* < 0.05 level and ROIS were hand-drawn using pycortex. Note therefore, that we opted to not use the pre-drawn definitions packaged with the NSD dataset, which were defined according to a liberal *t* > 0 thresholding. Our definition of the EBA overlaps with the ref. ^26^ atlas regions LO2, TO1 and TO2; thus, only the EBA is displayed on cortical flatmaps to avoid overplotting. The relationship between these regions is shown in Extended Data Fig. 6a.

### Statistical testing

#### Topographic connectivity scores

To generate a measure of topographic connectivity, the out-of-set *R*^2^ values for visual and somatosensory connective-field predictions were corrected for the *R*^2^ of a non-topographic null model, of which the predictions were generated through the mean V1 and S1 time courses, respectively. This means that, although the corrected values are no longer interpretable as variance explained, they reflect the superiority of the generalization performance of a spatial connective-field model relative to a non-spatial model. This correction therefore conservatively assesses the presence of true topographic connectivity by referencing against an explicit null model. One-sample *t*-tests were conducted to compare these corrected scores against zero and reported *P* values are two sided. To provide estimates of the magnitude of topographic connectivity, we computed the effect size Cohen’s *d*_*z*_ through the formula:

$${d}_{z}=\frac{t}{\sqrt{N}}$$

The within-participant differences in topographic connectivity scores were analysed using repeated-measures ANOVA, implemented in the afex package in the R programming language^63^. In modelling pairwise differences in topographic connectivity scores between ROIS, we performed Holm–Bonferroni correction of *P* values to account for the number of tests conducted. This was implemented using the emmeans R package^64^. We note that, given our *n* (174) and alpha level (*α* = 0.05), the analyses described are powered to detect a Cohen’s *d*_*z*_ in excess of 0.149, indicating that only very small effect sizes could remain undetected by such tests.

#### Thresholding of model performance

To distinguish between signal and noise in our parameter estimates, we used several strategies to threshold according to model performance. For visualization and analysis of group-level results (Fig. 1e), we thresholded according to vertex locations that have significant topographic connectivity according to a one sample *t*-test (see above). Any areas in which the group-level topographic connectivity is significantly greater than 0 are shown on the plot (*α* = 0.05). This measure is more conservative than *R*^2^, as it is a cross-validated performance measure explicitly referenced against a null model. We note that this thresholding procedure yields regions previously defined as being somatotopic and parameter estimates from these regions agree well with conventionally defined estimates derived from exogenous stimulation (Extended Data Fig. 5e–h).

Moreover, all somatotopic regions analysed in this manuscript survived multiple-comparisons correction using threshold-free cluster enhancement (TFCE), which controls for family-wise error while accounting for spatial correlations in the data^65^. Rather than applying an initial cluster-forming threshold, TFCE integrates evidence for cluster-like structure across all possible thresholds by weighting both the height of the statistic and the spatial extent of signal support. Specifically, topographic connectivity scores were first converted into TFCE scores, which were then compared against a null distribution generated by random sign-flips across participants. For each permutation, the TFCE transformation was recomputed and the maximum TFCE value retained, yielding a null distribution of 2,000 maximum scores. The observed TFCE scores were then conservatively evaluated against this null distribution, with corrected *P* values derived from the corresponding quantiles (*α* = 0.01, two-tailed).

For individual-participant outcomes, we used procedures that mirrored those of the HCP 7 T retinotopy pipeline^66^. For each modality-split variance-explained score (visual, somatosensory), we determined a threshold by fitting a Gaussian mixture model with two Gaussians to the distribution of variance explained values across vertices (excluding source regions) and then identified the value at which the posterior probability switches from the Gaussian with the lower mean to that with the higher mean. The interpretation of this procedure is that the Gaussian with a lower mean is likely to reflect noise (vertices that are not responsive to visual or somatosensory information), the Gaussian with larger mean is likely to reflect signal (vertices that are sensitive to visual or somatosensory information) and values above the threshold are more likely to reflect signal than noise. This procedure resulted in a visual variance explained threshold of 2.4% and a somatosensory variance explained threshold of 1.9%, which are very close to those used for the HCP retinotopy data (2.2%). The application of this procedure to the HCP average participant yielded a threshold of 10% for the somatosensory modality.

To evaluate the consistency in sensitivity to visual and somatosensory information at the individual level, we summed the number of participants for whom both modalities were above threshold at each vertex location (*N*^vs^). To statistically evaluate the likelihood of obtaining the observed *N*^vs^ under a null hypothesis (no systematic co-localization of tuning across individuals), we performed a permutation-based cluster analysis. First, we decomposed the cortical surface for each hemisphere into 400 LBOEs and used these spatial profiles as a design matrix for a regression model that predicted the presence of above threshold somatosensory tuning at each cortical location (0 = below threshold, 1 = above threshold). We next used the resulting *β* weights to predict 1,000 new, surrogate maps of somatosensory tuning by randomizing their sign prior to the dot product with the design matrix. The resulting maps therefore had the same spatial frequency profile as the empirical data but with randomized structure in cortical space.

For each surrogate somatosensory map, we calculated the *N*^vs^ at each vertex location and submitted this to a cluster analysis, whereby clusters were defined as contiguous sets of vertices with at least 96 participants above threshold for both modalities at that location (upper binomial limit for *n* = 174, *α* = 0.05). We then retained the maximum cluster-wise summed *N*^vs^ as a test statistic and concatenated these across surrogate maps to form a null distribution. The same clustering procedure was performed on the empirical data and *P* values were obtained by calculating the proportion of the null distribution that was lower than the summed *N*^vs^ in each empirical cluster.

The resulting data are shown in Extended Data Fig. 7a,b. Four bilateral clusters were detected, the largest of which encompassed dorsolateral visual cortex and parts of the superior parietal lobule. Other, smaller clusters were observed in the superior temporal lobe and frontally, including one overlapping with the frontal eye field. The profile of *N*^vs^ also revealed a small ‘hotspot’ of reliably above-threshold tuning in posterior parietal cortex, which may correspond to the visuotactile map described previously^28^ (Extended Data Fig. 7b–e).

#### Connective-field sampling extent

The approach reported here differs from classical connective-field estimation of a Gaussian centre (*V*_0_) and an explicit size (*σ*) parameter. Our model removes this constraint and allows flexible estimation of more complex spatial patterns, requiring us to develop a metric to approximate sampling extent.

To provide such estimates, we calculated the median geodesic distance from the peak of each connective-field profile at which it is above its half-maximum. Note that such a computation ignores data from the opposite hemisphere to the peak, as cortical hemispheres are not contiguous surfaces. These quantifications are shown in Extended Data Fig. 10. When calculating this metric for V1 connective fields, we observe coherent increases in V1 sampling extent with distance from V1 that mirror those derived from classical connective-field models/pRF mapping^12^ and are consistent with the sizes expected from previous video-watching-derived estimates^15^.

Similarly, for S1 connective fields, we observed a strong positive relationship between S1 sampling extent and geodesic distance from S1 in frontal and parietal directions, with weaker relationships in the medial and insular directions. This pattern of results aligns well with somatotopic mapping studies^67^, which demonstrate hierarchical-gradient-like decreases in bodily selectivity. This method of estimating connective-field sampling extent is validated by mirroring organizational hallmarks consistent with previous empirical studies.

#### Cortical coverage of somatotopic connectivity and relation to functional localizer

The extent of cortical coverage was assessed via a standard bootstrapping procedure. Individual-participant topographic connectivity scores were resampled with replacement 10,000 times to generate resampled datasets with random samples of participants. For each resampled dataset, we quantified the percentage of voxels within each somatosensory ROI with topographic connectivity scores significantly greater than zero. In this calculation, note that the source region of the analysis (3b) is excluded. Reported CIs were obtained from the quantiles (2.5% and 97.5%) of the resulting distribution of percentage coverage estimates. The same bootstrapping approach was applied to assess the correlation of somatotopic connectivity scores with functional localizer data from the NSD dataset, for which we used the same group-level *t*-statistics described above. Using the correlation between somatotopic connectivity scores and the ‘body v all other categories’ *t*-statistics as a reference, we subtracted the correlation with the corresponding place, face and object *t*-statistics across 10,000 bootstrapped samples. The resulting distributions of correlation differences were used to compute *P* values.

#### Robustness of extrastriate somatotopic maps: permutation test

To evaluate the robustness of extrastriate somatotopic maps, we tested the null hypothesis that out of set somatotopic maps are predictable from maps generated from randomized connective fields, but with preserved autocorrelation structure. Referencing the out-of-set prediction of empirical maps against surrogate instances is important, as the spatial autocorrelation inherent in brain data implies that spatially proximal measurements are likely to be similar, regardless of how they were derived^68^. As such, the statistical significance of agreement between maps is likely to be inflated and violate independence assumptions. Thus, rather than relying on the statistical significance of these empirical agreement statistics alone, they require benchmarking against surrogate data that quantifies the statistical expectations under a null hypothesis.

To this end, we first used the somatotopic map estimated for the cutout region in Fig. 4a from one split-half of participants to predict the corresponding data from the other split-half. The resulting *R*^2^ value was retained as an empirical test statistic. To generate a null distribution of these statistics, we generated 10,000 ‘surrogate’ homuncular maps from each split-half of participant data. These were generated by taking the estimated b* corresponding to each of the LBOEs of the S1 subsurface, randomizing their sign and recomputing the S1 connective field. This manipulation generates randomized connective fields that preserve the same amplitude spectra (distribution of energy across frequency) as the empirical data. For each surrogate dataset, we then computed its performance in predicting the out of set empirical somatotopic map and retained the proportion of these statistics that exceeded the empirical value as a *P* value. This process was repeated for the second participant split and the resulting *P* values were summed to obtain a final measure of the probability of obtaining the empirical statistic under the null hypothesis.

#### Visual body-part selectivity estimates

To estimate a map of visual body-part selectivity, we leveraged data from the NSD, also collected at 7 T (ref. ^69^). Specifically, we used the denoised single trial *β*-estimates from the final 12 runs of data of all 8 participants. This corresponded to 9,000 functional volumes, each of which included voxel-wise estimates of the response to an image from the Common Objects in Context (COCO) dataset^70^. Using Connectome Workbench commands, the cortex-wide single-trial *β*-estimates were nearest-neighbour resampled from fsaverage space to the same 59,000 vertex-per-hemisphere surface format as the HCP data.

We next used a corpus dataset of estimated body-part keypoints within each image of the COCO dataset, which were generated by Openpose^71^, a convolutional neural network based pose-estimation toolkit. Openpose detects the location of 17 different keypoints (nose, left eye, right eye, left ear, right ear, left shoulder, right shoulder, left elbow, right elbow, left wrist, right wrist, left hip, right hip, left knee, right knee, left ankle, right ankle). Thus, each image in the COCO dataset is associated with 17 binary variables that codes the presence of each keypoint for every human entity within the image. Critically, the subset of COCO images used in the NSD dataset were spatially cropped relative to the original versions for which the keypoints were computed. We therefore recoded keypoints that were coded as present in the original COCO images but resided outside of the NSD crop-box as being 0 (absent). For each image and body part, we calculated the average of the binary variable for each keypoint across entities to give an estimate of the frequency with which the body part was present within entities within the image.

Next, we converted these scores into a regressor that explicitly coded selective responses for each body part. For each body part, we calculated a selectivity score defined as follows:

$${X}_{{\rm{B}}{\rm{o}}{\rm{d}}{\rm{y}}{\rm{P}}{\rm{a}}{\rm{r}}{\rm{t}}}={\rm{B}}{\rm{o}}{\rm{d}}{\rm{y}}{\rm{P}}{\rm{a}}{\rm{r}}{\rm{t}}{\rm{P}}{\rm{r}}{\rm{e}}{\rm{s}}{\rm{e}}{\rm{n}}{\rm{c}}{\rm{e}}\times ({N}_{{\rm{B}}{\rm{o}}{\rm{d}}{\rm{y}}{\rm{P}}{\rm{a}}{\rm{r}}{\rm{t}}{\rm{A}}{\rm{b}}{\rm{s}}{\rm{e}}{\rm{n}}{\rm{c}}{\rm{e}}})$$

In this calculation, for example, an image with the presence of an ankle and the absence of all other body parts generates the highest score for the ankle selectivity regressor. Conversely, the score will be implicitly penalized as a function of the number of other (non-ankle) body parts that are visible. These regressors for each body part were stacked into a design matrix. This formed the basis of a forward model of visual body selectivity, of which the parameters were estimated by ridge regression. The model was trained on 10 of the runs of data through *k*-fold cross-validation and was tested on the final 2. For each voxel, body part preference was defined as the dot product of the resulting *β* weights and their ordinal position in the S1 homunculus (ankle to nose) providing a continuous map of visual body part selectivity along a similar toe–tongue axis as the somatotopy data.

#### Searchlight analyses: permutation test

Within the cutout region in Fig. 4 we defined geodesic ‘chunks’ of cortex that were centred at each vertex with a radius of 8 mm. To generate empirical statistics, within each one of these chunks we computed the correlation between the somatotopic map shown in Fig. 4 and the target data (vertical visual field position in retinotopic map or visual body part selectivity map). To generate a null distribution of these statistics, we leveraged the same 10,000 phase-scrambled surrogate homuncular maps (see the ‘Robustness of extrastriate somatotopic maps: permutation test’ section) and, for each one, we calculated the corresponding correlations with the target data in each chunk. This distribution of local chunk-wise correlations obtained across all surrogate somatotopic maps served as our null distribution of local correlations. *P* values for the correlations within each empirical chunk were obtained as the probability of obtaining such extreme statistics from this null distribution. Note that, for the analysis of the retinotopy data, we excluded chunks for which the range of the estimated visual field positions was less than 1 DVA and therefore had little retinotopic variation. The resulting *P* values were then projected into the spatial locations of their corresponding chunk and the data were then averaged across hemispheres to produce the data in Fig. 5e,i. Note that as the chunks contained partially overlapping data, the data in Fig. 5 show the lowest *P* value obtained at each vertex location. In addition to the results reported in the main text, we performed additional analyses that correlated the eccentricity parameter of the retinotopy data with the somatotopic map. This revealed a region overlapping with IPS0 and IPS1 wherein more foveal locations were tuned to facial features and more eccentric locations tuned to limbs (Extended Data Fig. 8).

### Alternative model: field-based connective-field model

There is evidence that S1 is not a continuous somatotopic map, but consists of discrete body-part fields, defined on the basis of resting state connectivity, myelin and functional data^17^. Accordingly, a model that assumes discontinuous connectivity at the boundary of such fields, rather than continuous connectivity along the 3b surface, may provide a viable alternative account of the data. To explicitly test this organizing influence of body part fields, we compared our full connective-field model that uses all of 3b as a source region to 4 alternative connective field models (Extended Data Fig. 8e), each with restricted source regions corresponding to the lower limb, trunk, upper limb and face fields, following the definitions described in the ‘Analysis of subfields within the S1’ section. To ensure validity of such a comparison, we determined the number of LBOEs in each of these restricted source regions sufficient to reconstruct 4 mm Gaussian connective fields at near-ceiling performance (determined by the minimum *R*^2^ being above 0.98 across vertex locations). This implied 70 LBOEs each for the lower limb, trunk and face fields, and 50 for the upper limb field. These alternative models were then fit using identical procedures described for the full model. For each vertex location, we then selected the cross-validated somatosensory *R*^2^ from the best performing restricted model and subtracted it from that of the full model, yielding a Δ*R*^2^. These outcomes are shown in Fig. 4e,f.

### Reporting summary

Further information on research design is available in the Nature Portfolio Reporting Summary linked to this article.

## Online content

Any methods, additional references, Nature Portfolio reporting summaries, source data, extended data, supplementary information, acknowledgements, peer review information; details of author contributions and competing interests; and statements of data and code availability are available at 10.1038/s41586-025-09796-0.

## Supplementary information

Reporting Summary

## Data Availability

7 T resting-state and video-watching data from the human connectome project are available on the human connectome project website (https://www.humanconnectome.org/study/hcp-young-adult) pending compliance with the WU-Minn HCP Consortium Open Access Data Use Terms (https://www.humanconnectome.org/study/hcp-young-adult/document/wu-minn-hcp-consortium-open-access-data-use-terms). Data from the NSD are available from Amazon Web Services (https://registry.opendata.aws/nsd/). Data from the whole-body somatotopy dataset are available at the OpenNeuro repository (https://openneuro.org/datasets/ds004044, 10.18112/openneuro.ds004044.v2.0.3). Data from the COCO dataset are available online (https://cocodataset.org/#download).

## Code availability

Code for performing the analyses described in this Article is maintained at GitHub (https://github.com/N-HEDGER/Vicarious_somatotopy).
