# Supplementary material for: Vicarious body maps bridge vision and touch in the human brain
Source: Nature. 2025 Nov 26;650(8100):173–81. doi: 10.1038/s41586-025-09796-0 (PMC12872459; doi:10.1038/s41586-025-09796-0)
Supplement: Supplementary file 1 — Reporting Summary [file 41586_2025_9796_MOESM1_ESM.pdf]

nature portfolio

Corresponding author(s): Nicholas Hedger, Tomas Knapen

Last updated by author(s): 13.10.2025

Reporting Summary

Nature Portfolio wishes to improve the reproducibility of the work that we publish. This form provides structure for consistency and transparency in reporting. For further information on Nature Portfolio policies, see our Editorial Policies and the Editorial Policy Checklist.

Statistics

For all statistical analyses, confirm that the following items are present in the figure legend, table legend, main text, or Methods section.

n/a	Confirmed	
☐	☒	The exact sample size (*n*) for each experimental group/condition, given as a discrete number and unit of measurement
☐	☒	A statement on whether measurements were taken from distinct samples or whether the same sample was measured repeatedly
☐	☒	The statistical test(s) used AND whether they are one- or two-sided<br>*Only common tests should be described solely by name; describe more complex techniques in the Methods section.*
☐	☒	A description of all covariates tested
☐	☒	A description of any assumptions or corrections, such as tests of normality and adjustment for multiple comparisons
☐	☒	A full description of the statistical parameters including central tendency (e.g. means) or other basic estimates (e.g. regression coefficient) AND variation (e.g. standard deviation) or associated estimates of uncertainty (e.g. confidence intervals)
☐	☒	For null hypothesis testing, the test statistic (e.g. *F*, *t*, *r*) with confidence intervals, effect sizes, degrees of freedom and *P* value noted<br>*Give P values as exact values whenever suitable.*
☐	☒	For Bayesian analysis, information on the choice of priors and Markov chain Monte Carlo settings
☐	☒	For hierarchical and complex designs, identification of the appropriate level for tests and full reporting of outcomes
☐	☒	Estimates of effect sizes (e.g. Cohen's *d*, Pearson's *r*), indicating how they were calculated

Our web collection on statistics for biologists contains articles on many of the points above.

Software and code

Policy information about availability of computer code

Data collection

No software was used to collect the data for this study since it is analysis of secondary data.

Data analysis

Custom scripts used to perform the analyses associated with this paper are available at this reporsitory https://github.com/N-HEDGER/Vicarious_somatotopy. Required packages are listed in the repository but are repeated here:

requests
nibabel
pyyaml
himalaya==0.3.5
cifti==1.1
torch==2.1.1
numpy==1.26.2
tqdm
scikit-learn==1.3.2
matplotlib==3.8.2
pycortex
mne==1.9.0

R packages used to perform analyses include:

emmeans==1.11.1

afex==1.4.1

For manuscripts utilizing custom algorithms or software that are central to the research but not yet described in published literature, software must be made available to editors and reviewers. We strongly encourage code deposition in a community repository (e.g. GitHub). See the Nature Portfolio guidelines for submitting code & software for further information.

Data

Policy information about availability of data

All manuscripts must include a data availability statement. This statement should provide the following information, where applicable:

- Accession codes, unique identifiers, or web links for publicly available datasets
- A description of any restrictions on data availability
- For clinical datasets or third party data, please ensure that the statement adheres to our policy

All data analyzed in this manuscript are freely available from the following sources

7T resting-state and movie watching data from the human connectome project are available on the human connectome project website (https://www.humanconnectome.org/study/hcp-young-adult) pending compliance with the WU-Minn HCP Consortium Open Access Data Use Terms https://www.humanconnectome.org/study/hcp-young-adult/document/wu-minn-hcp-consortium-open-access-data-use-terms).

Data from the natural scenes dataset are available from Amazon Web Services at https://registry.opendata.aws/nsd/.

Data from the whole body somatotopy dataset are available at the OpenNeuro repository at https://openneuro.org/datasets/ds004044 (doi:10.18112/openneuro.ds004044.v2.0.3)

Data from the COCO dataset are available from https://cocodataset.org/#download

Research involving human participants, their data, or biological material

Policy information about studies with human participants or human data. See also policy information about sex, gender (identity/presentation), and sexual orientation and race, ethnicity and racism.

Reporting on sex and gender	Please note that for the ensuing sections on demograpic information that only limited information is available without access to 'restricted' HCP data.<br><br>Gender: 70 males, 104 females.
Reporting on race, ethnicity, or other socially relevant groupings	Born in Missouri, USA. 88.5% of the sample identified as "White" (4% Asian, Hawaiian or other pacific Aslan), 6.3% black or African American, 1.1% unreported.
Population characteristics	N/A
Recruitment	The 7 Tesla (7T) Human Connectome Project (HCP) dataset includes high-resolution MRI scans from 184 healthy young adults, aged 22 to 35. These participants were primarily recruited from the existing pool of subjects involved in the HCP's 3T imaging studies. The recruitment strategy focused on individuals who had already contributed to the 3T dataset, ensuring consistency and comparability across different imaging modalities.<br><br>The HCP aimed to recruit large groups of directly related siblings, referred to as "sibships," ideally including four or more brothers or sisters. This approach was designed to facilitate heritability analyses and genetic association studies. The recruitment process targeted a diverse demographic, including White non-Hispanic, Hispanic, Asian, and African-American families, to reflect the ethnic diversity of the United States.<br><br>The HCP utilized the Missouri Family Registry, a resource developed by Washington University in St. Louis, to identify and contact potential participants. This registry provided a database of families suitable for the study's criteria.<br><br>Potential participants underwent telephone screening interviews to assess eligibility. The HCP team developed methods to encourage participation, including providing detailed information about the study's purpose and procedures.<br><br>More detail is provided on the HCP website: http://www.humanconnectome.org
Ethics oversight	Washington University Institutional Review Board (IRB) (approval number 201204036).

Note that full information on the approval of the study protocol must also be provided in the manuscript.

Field-specific reporting

Please select the one below that is the best fit for your research. If you are not sure, read the appropriate sections before making your selection.

☒ Life sciences ☐ Behavioural & social sciences ☐ Ecological, evolutionary & environmental sciences

For a reference copy of the document with all sections, see nature.com/documents/nr-reporting-summary-flat.pdf

Life sciences study design

All studies must disclose on these points even when the disclosure is negative.

Sample size	174 human participants. Note that these data derive from a secondary data source (the human connectome project) and hence we did not perform an a-priori power calculation (the data are already collected). As noted in our methods section - given our N (174) and alpha level (α = .05) the analyses described in our manuscript are powered to detect a Cohen's dz in excess of 0.149 - indicating only very small effect sizes could remain undetected by such tests.
Data exclusions	Functional / anatomical preprocessing did not produce adequate results in 7 participants - and so 7 of an original 181 participants were analyzed.
Replication	We assess replicabiliity of model parameters via cross-validation on independent test set data and performing model fitting on 2 independent subject folds. We find parameters are highly consistent between these folds, pointing to the robust nature of the findings.
Randomization	N/A no group allocation across subjects, within subjects all cross validation was performed exhaustively
Blinding	N/A no group allocation

Reporting for specific materials, systems and methods

We require information from authors about some types of materials, experimental systems and methods used in many studies. Here, indicate whether each material, system or method listed is relevant to your study. If you are not sure if a list item applies to your research, read the appropriate section before selecting a response.

Materials & experimental systems

n/a	Involved in the study	
☒	☐	Antibodies
☒	☐	Eukaryotic cell lines
☒	☐	Palaeontology and archaeology
☒	☐	Animals and other organisms
☒	☐	Clinical data
☒	☐	Dual use research of concern
☒	☐	Plants

Methods

n/a	Involved in the study	
☒	☐	ChIP-seq
☒	☐	Flow cytometry
☐	☒	MRI-based neuroimaging

Plants

Seed stocks	NA
Novel plant genotypes	NA
Authentication	NA

Magnetic resonance imaging

Experimental design

Design type	Passive free-viewing movie watching and resting state

Design specifications

Participants were scanned while watching short (ranging from 1 to 4.3 minutes in length) independent and Hollywood film clips that were concatenated into movies of 11.9 - 13.7 minutes total length. Before each clip, and after the final clip was displayed, there were 20 second 'rest' periods wherein there was no auditory stimulation and only the word 'REST' presented on the screen. There were 4 separate functional runs, wherein observers viewed 4 separate movies. All 4 movies contained an identical 83 second 'validation' sequence at the end of the movie.

Participants also took part in one hour of resting state scans, also split into 4 runs of equal (~15 min) length. Full details of the procedure and experimental setup are reported in the HCP S12000 release reference manual.

Behavioral performance measures

N/A

Acquisition

Imaging type(s)

Functional and anatomical

Field strength

7T

Sequence & imaging parameters

Sequence: gradient echo EPI, TR: 1000 ms, TE: 22.2ms, flip angle: 45 deg, FOV: 208 x 208 mm (RO x PE), matrix: 130 x 130 (RO x PE), slice thickness (1.6mm, 85 slices, 1.6mm isotropic voxels), Multiband factor: 5, Image Acceleration factor (iPAT): 2, partial fourier sampling 7 / 8, echo spacing: 0.64ms, BW: 1924 Hz/Px

Area of acquisition

Whole-brain

Diffusion MRI ☐ Used ☒ Not used

Preprocessing

Preprocessing software

The preprocessing pipeline and custom software is described in detail in the HCP reference manual and the routines are available here: https://github.com/Washington-University/HCPpipelines

Normalization

Achieved via the MSMAII alignment method. This is described in "A multi-modeal parcellation of human cerebral cortex (Glasser, Nature , 2016) and the routines are available here: https://github.com/Washington-University/HCPpipelines

Normalization template

MNI 152 for subcortex, 59k vertex per hemisphere average subject surface for cerebral cortex.

Noise and artifact removal

FIX ICA based denoising, after which we performed high pass filtering (Savitzky Golay filter (3rd order, 210 seconds in length),) and % signal change conversion.

Volume censoring

NA

Statistical modeling & inference

Model type and settings

(The below is copied from our methods section. Note that formulas, figures and other details are best explained in this methods section, since they will not render in this form.)

All model fitting was conducted in python, exploiting the routines implemented by the 'Himalaya' package. Here, we employed banded ridge regression, which belongs to a family of 'regularized' regression techniques that estimate a regularization parameter λ to improve generalisation performance. Banded ridge regression expands on these techniques by estimating separate λi for separate feature spaces i of the design matrix X - thereby optimizing regularization strengths independently for each feature space. Banded ridge regression therefore respects the fact that different feature spaces in the design matrix may differ in covariance structure, number of features and prediction performance - entailing different optimal regularization.

In the present case, our two feature spaces consisted of the visual and somatosensory modalities, or equivalently, the 400 V1 and S1 model timecourses (Xv1, Xs1) described in the previous section. Thus, to model brain activity of a particular voxel, banded-ridge regression computes the weights b*i, as defined below:

(see formula in methods)

Similarly to un-banded ridge regression, the ridge weights b*i are estimated from the training data and the hyperparameters λi are learned via cross validation. In the present case, our training data consisted of four runs of functional data wherein participants watched an independent movie. This natural organization of the data allowed us to use a leave one movie out cross-validation strategy to estimate λi.

Effect(s) tested

At each voxel location we test the superiority of our connective field models to that of a non-spatial null model. We additionally test for pairwise comparisons between conditions and ROIs via holm-corrected pairwise t tests. These procedures are detailed at length in the methods section.

Specify type of analysis: ☐ Whole brain ☐ ROI-based ☒ Both

Anatomical location(s)

> As described in the manuscript, we leverage various regions of interest from the HCP multimodal parcellation: Glasser, M. F. et al. A multi-modal parcellation of human cerebral cortex. Nature 536, 171–178 (2016).
> Wang retinotopy atlas: Wang, L., Mruczek, R. E., Arcaro, M. J. & Kastner, S. Probabilistic Maps of Visual Topography in Human Cortex. Cereb Cortex 25, 3911–3931 (2015).
> NSD floc-defined ROIs: Allen, E. J. et al. A massive 7T fMRI dataset to bridge cognitive neuroscience and artificial intelligence. Nat Neurosci 25, 116–126 (2022).
> and an atlas of temporal visual cortex: Rosenke, M., van Hoof, R., van den Hurk, J., Grill-Spector, K. & Goebel, R. A Probabilistic Functional Atlas of Human Occipito-Temporal Visual Cortex. Cerebral Cortex 31, 603–619 (2021).

Statistic type for inference

(See Eklund et al. 2016)

Vertex and ROI-wise

Correction

Holm-Bonferroni based correction for ROI-based tests. For one sample tests of model performance against zero (see above) we present these surface-level results without correction for multiple comparisons, but note that all regions analyzed further survive cluster-based correction without smoothing (see “thresholding of model performance”) via cluster-based correction using threshold free cluster enhancement (TFCE, Smith and Nichols, 2009). TFCE is a robust method because it avoids the need to select an arbitrary initial cluster-forming threshold and instead integrates evidence for both signal strength and spatial extent across all possible thresholds.

Models & analysis

n/a	Involved in the study
☒	☐ Functional and/or effective connectivity
☒	☐ Graph analysis
☒	☐ Multivariate modeling or predictive analysis
